# Meta‐analysis of gonadal transcriptome provides novel insights into sex change mechanism across protogynous fishes

**DOI:** 10.1111/gtc.13166

**Published:** 2024-09-29

**Authors:** Ryo Nozu, Mitsutaka Kadota, Masaru Nakamura, Shigehiro Kuraku, Hidemasa Bono

**Affiliations:** ^1^ Laboratory of Genome Informatics, Graduate School of Integrated Sciences for Life Hiroshima University Higashi‐Hiroshima Japan; ^2^ Laboratory of BioDX, Genome Editing Innovation Center Hiroshima University Hiroshima Japan; ^3^ Laboratory for Phyloinformatics RIKEN Center for Biosystems Dynamics Research (BDR) Kobe Japan; ^4^ Laboratory for Developmental Genome System RIKEN Center for Biosystems Dynamics Research (BDR) Kobe Japan; ^5^ Okinawa Churashima Research Center Okinawa Churashima Foundation Motobu‐cho Japan; ^6^ Molecular Life History Laboratory, Department of Genomics and Evolutionary Biology National Institute of Genetics Mishima Japan; ^7^ Department of Genetics Graduate University for Advanced Studies, SOKENDAI Mishima Japan

**Keywords:** genome, gonad, meta‐analysis, protogynous fishes, sex change, transcriptome

## Abstract

Protogyny, being capable of changing from female to male during their lifetime, is prevalent in 20 families of teleosts but is believed to have evolved within specific evolutionary lineages. Therefore, shared regulatory factors governing the sex change process are expected to be conserved across protogynous fishes. However, a comprehensive understanding of this mechanism remains elusive. To identify these factors, we conducted a meta‐analysis using gonadal transcriptome data from seven species. We curated data pairs of ovarian tissue and transitional gonad, and employed ratios of expression level as a unified criterion for differential expression, enabling a meta‐analysis across species. Our approach revealed that classical sex change‐related genes exhibited differential expression levels between the ovary and transitional gonads, consistent with previous reports. These results validate our methodology's robustness. Additionally, we identified novel genes not previously linked to gonadal sex change in fish. Notably, changes in the expression levels of acetoacetyl‐CoA synthetase and apolipoprotein Eb, which are involved in cholesterol synthesis and transport, respectively, suggest that the levels of cholesterol, a precursor of steroid hormones crucial for sex change, are decreased upon sex change onset in the gonads. This implies a potential universal influence of cholesterol dynamics on gonadal transformation in protogyny.

## Graphical abstract

The meta‐analysis across multiple fish species confirmed differential expression of classical sex change‐related genes. Novel sex change‐associated genes, including those linked to cholesterol, were identified. Cholesterol dynamics may universally influence sex change across protogynous fishes.

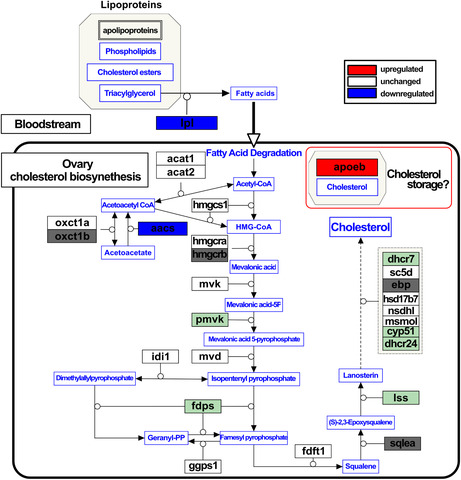

## 1 INTRODUCTION

**FIGURE 1:**
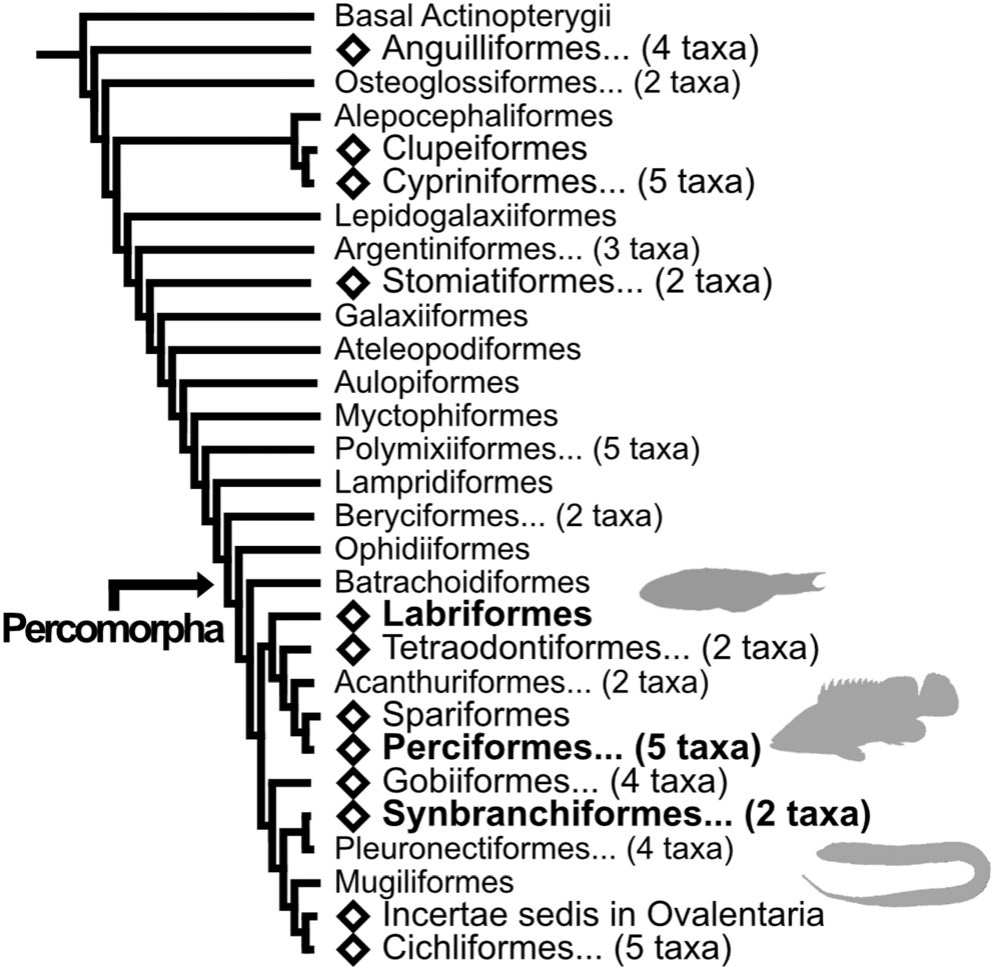
Summarized phylogenetic tree of Actinopterygii. The order‐level phylogenetic tree is shown. The phylogenetic tree was derived from Rabosky et al. (2018) and modified. Diamonds indicate orders with sequential hermaphroditism. Numbers in parentheses indicate the number of summarized orders. Orders containing the fish species used in this study are shown in bold.

Sequential hermaphroditism, a phenomenon in which individuals undergo sex change during their lifetime, has been extensively recognized in teleosts over the past several decades (Atz, 1964). Sequential hermaphroditism has been categorized into three major types based on the direction of sex change (protogyny, where females change to males; protandry, where males change to females; and bidirectional sex change, where individuals can change both sexes serially). Phylogenetic analysis has shown that sequential hermaphroditism has been acquired in various evolutionary lineages (Mank et al., 2006) (Figure 1). Interestingly, protogyny is prevalent in 20 families but is thought to have emerged once in evolutionally the Percomorpha (Kuwamura et al., 2020). Therefore, there are common features in the intricate regulatory mechanisms during protogynous sex change. Specifically, the replacement of ovarian tissue with testicular tissue is commonly observed during protogynous sex change (Bhandari et al., 2003; Lo Nostro et al., 2003; Muncaster et al., 2013; Nakamura et al., 1989; Ohta et al., 2008). Additionally, considering the high functional conservation of gonads across organisms, the existence of a regulatory mechanism governing gonadal transformation comprising common and essential factors across protogynous fishes is plausible; however, a comprehensive perspective of this process remains elusive.

Accordingly, elucidation of the molecular mechanisms underlying the gametogenesis switch in sex‐changing fish has subsequently become a focus of research. In particular, the expression patterns of multiple sex‐related genes during sex change within the family Labridae, which serves as a model for protogynous sex change, have been explored (Horiguchi et al., 2018; Miyake et al., 2012; Thomas et al., 2019). Recently, next‐generation sequencing technology has enabled researchers to conduct comprehensive analyses of gene expression, allowing the identification of global gene expression profiles during gonadal transformation in protogynous fish belonging to Labriformes, Perciformes, and Synbranchiformes (Fan et al., 2022; He et al., 2022; Senthilkumaran et al., 2017; Todd et al., 2019; Wu et al., 2020). Thus, these results suggest the involvement of several pivotal genes in the ovary‐to‐testis transformation in sex‐changing fish.

For example, a repertoire of sex‐related genes, including cytochrome P450, family 19, subfamily A, polypeptide 1a (*cyp19a1a*), forkhead box L2a (*foxl2a*), folliculogenesis specific bHLH transcription factor (*figla*), anti‐Müllerian hormone (*amh*), gonadal somatic cell derived factor (*gsdf*), doublesex and mab‐3 related transcription factor 1 (*dmrt1*) were identified as differentially expressed genes (DEGs) during sex change across protogynous fishes. Notably, the gonadal expression of *cyp19a1a* and *foxl2a*, which are key players in maintaining ovarian status, is reduced upon the onset of sex change in multiple species (Li et al., 2006; Thomas et al., 2019; Zhang et al., 2013). *cyp19a1a* encodes aromatase, an enzyme responsible for the conversion of androgens (e.g., testosterone) to estrogens (e.g., estradiol‐17ß; E2), whereas *foxl2a* encodes a transcription factor that targets and up‐regulates *cyp19a1a* in teleost fishes (Wang et al., 2007; Zhang et al., 2022). Furthermore, the initiation of female‐to‐male sex change, triggered by a rapid decline in E2 levels (Nakamura et al., 1989), is well‐coordinated with the expression profiles of these genes. Conversely, *amh*, *gsdf*, and *dmrt1* are crucial for testicular differentiation and development in teleost fishes (Kobayashi et al., 2008; Rodríguez‐Marí et al., 2005; Shibata et al., 2010; Skaar et al., 2011; Wang & Orban, 2007; Webster et al., 2017; Zhang et al., 2016). In several sex‐changing species, the upregulation of these genes coincides with the emergence of testicular tissue during transition, suggesting that these genes are involved in testicular formation during gonadal sex change (Horiguchi et al., 2013; Horiguchi et al., 2018; Nozu et al., 2015; Thomas et al., 2019; Zhu et al., 2016).

Transcriptome analysis during gonadal transformation has also revealed differential expression of several genes not previously implicated in sex determination or differentiation pathways. In particular, upregulation of apolipoprotein Eb (*apoeb*), ectonucleotide pyrophosphatase/phosphodiesterase 2 (*enpp2*), and l‐rhamnose‐binding lectin CSL2 (*csl2*) expression, accompanied by downregulation of galectin‐3 (*gal3*), was observed during the early phase of gonadal sex change in the protogynous Asian swamp eel *Monopterus albus*. These findings suggest that these genes are involved in gonadal transformation, particularly during the initiation of sex change (Fan et al., 2022; Senthilkumaran et al., 2017). However, verifying their direct involvement and commonality in the sex change process remains challenging.

Recently, by amalgamating transcriptome data from multiple studies, meta‐analysis has attracted attention as a powerful tool capable of providing novel insights overlooked by traditional hypothesis‐driven research methods (Bono, 2021; Rahman et al., 2020; Toga & Bono, 2023). In other words, it is expected that by reanalyzing studies across multiple species and studies, universal factors controlling gonadal transformation, which have not been detected in individual studies, could be identified among protogynous fishes. Therefore, the objective of the present study was to identify the shared factors involved in gonadal transformation across species using a meta‐analysis of publicly available transcriptome data from multiple experiments and species. Furthermore, in this study, we focused on another well‐studied species, the three‐spot wrasse, *Halichoeres trimaculatus*, a protogynous species belonging to the family Labridae and produced de novo genome assembly and gonad transcriptome data undergoing sex change. This new data consolidated our dataset encompassing seven protogynous species from three orders allowing exhaustive transcriptome analysis. Through this dataset and uniform pipeline, we identified genes with differential expression levels between the transitional gonads and ovaries (Figure S1). We reevaluated the relationship between these genes and gonadal sex change, providing a novel perspective regarding the mechanisms underlying this process.

## 2 RESULTS

### 2.1 De novo genome assembly and transcriptome of gonadal tissue in the three‐spot wrasse

The genome sequencing was required for the RNA‐Seq analysis in this study, so the de novo genome assembly of the three‐spot wrasse, which had not been previously determined, was constructed. High‐throughput genome sequence data were obtained from a locally obtained female three‐spot wrasse from Okinawa, Japan. The obtained HiFi data, which comprised approximately 32 Gbp and encompassed approximately 1.9 million DNA sequences, were assembled using the hifiasm program (Cheng et al., 2021). Subsequently, the acquired Hi‐C data were used for scaffolding, resulting in the generation of 227 scaffolds. The longest scaffold measured 44.2 Mbp, with an N50 length (the shortest of the lengths covering 50% of the genome) of 37.9 Mbp, contributing to a total assembly length of approximately 849.9 Mbp. Assessment using BUSCO v5.4 (Manni et al., 2021) revealed a completeness >98%, indicating a high level of coverage and contiguity in the de novo genome assembly of the three‐spot wrasse (Table 1).

**TABLE 1:** Statistics of genome assembly in three‐spot wrasse (fHalTri1.1) and other Labidae fishes listed in NCBI genomes.

	fHalTri1.1	*Cheilinus undulatus* (humphead wrasse)	*Notolabrus celidotus* (New Zealand spotty)	*Labrus bergylta* (ballan wrasse)	*Semicossyphus pulcher* (California sheephead)	*Tautogolabrus adspersus* (cunner)
Assembly accession	BSYP01000001–BSYP01000227	GCF_018320785.1	GCF_009762535.1	GCF_900080235.1	GCA_022749685.1	GCA_020745685.1
Total seq length (Mbp)	849.9	1173.4	846.7	805.5	794.1	723.6
Max length (Mbp)	44.2	59.6	42.4	9.1	38.5	37.1
No. of scaffolds	227	45	467	13,466	178	347
Scaffold N50 (Mbp)	37.9	51.5	37.1	0.8	32.1	31.7
Scaffold L50	11	11	11	252	12	11
No. of seqs >1 Mbp	25	24	24	197	35	24
% of seqs >1 Mbp	97.3	99.7	97.6	43.9	97.1	99.9
No. of gaps	117	284	1133	257	8	283
Complete BUSCO (%)	98.7^a^	99.0	97.1	98.9	98.9	98.1

^a^ BUSCO version 5.4, reference datasets: vertebrata_odb10.

The RNA‐Seq reads obtained from transitional gonads and ovaries of additional females were mapped to the genome assembly, and the obtained mapping results were used to assemble the transcriptome for the annotation file (“merged.gtf”). This process yielded 27,573 transcript sequences (Table 2). Subsequently, 24,695 coding sequences (protein sequences) were predicted from the set of transcript sequences using TransDecoder (Haas, 2018).

**TABLE 2:** Statistics of orthology relationship among species.

Common name	Scientific name	Order	Reference genome	No. of transcripts seqs obtained based on the RNA‐seq data from gonadal tissue^a^	No. of protein seqs predicted by TransDecoder	No. of reciprocal best hit (vs. three‐spot wrasse)
Three‐spot wrasse	*Halichoeres trimaculatus*	Labriformes	Assembly in this study	27,573	24,965	24,965
Bluehead wrasse	*Thalassoma bifasciatum*	Labriformes	GCA_008086565.1	57,699	43,195	13,871
Hong Kong grouper	*Epinephelus akaara*	Perciformes	Ge et al., 2019	26,507	24,752	11,619
Orange‐spotted grouper	*Epinephelus coioides*	Perciformes	GCA_900536245.1	30,867	34,191	12,507
Black seabass	*Centropristis striata*	Perciformes	NA	95,941^b^	95,941	12,591
Asian swamp eel	*Monopterus albus*	Synbranchiformes	GCF_001952655.1	53,472	50,693	12,765
Zig‐zag eel	*Mastacembelus armatus*	Synbranchiformes	GCF_900324485.2	48,724	40,908	13,095

^a^ Based on the annotation file (merged gtf file), the extracted transcripts sequences from the genome by gffread.

^b^ Among transcriptome sequences (594,726 seqs) assembled by Trinity, transcripts sequences corresponding to coding seqences predicted via TransDecoder.

### 2.2 Data curation of public RNA‐Seq data and genomic information

We downloaded Sequence Read Archive (SRA) data from six fish species for which sex changes have been reported (Kuwamura et al., 2020): bluehead wrasse *Thalassoma bifasciatum* (Todd et al., 2019) (order Labriformes), orange‐spotted grouper *Epinephelus coioides* (Xiao et al., 2020) (order Perciformes), Hong Kong grouper *E. akaara* (Ge et al., 2019) (order Perciformes), black seabass *Centropristis striata* (Breton et al., 2019) (order Perciformes); Asian swamp eel *Monopterus albus* (Fan et al., 2022; Senthilkumaran et al., 2017; Zhao et al., 2018) (order Synbranchiformes), zig‐zag eel *Mastacembelus armatus* (Xue et al., 2021) (order Synbranchiformes). Furthermore, using the metadata associated with the SRA data, we curated the RNA‐Seq data into comparable sample pairs of intersexual (transitional) gonads and ovaries (a total of 31 paired data, including three‐spot wrasse). Table S1 presents the metadata, including Run accession numbers, for the RNA‐Seq data analyzed in this study.

The genome sequences of five of the seven species utilized in this study are publicly available (Table 2) and served as reference genomes for read mapping. The genome sequences of *T. bifasciatum* (GCA_008086565.1), *E. coioides* (GCA_900536245.1), *M. albus* (GCF_001952655.1), and *M. armatus* (GCF_900324485.2) were retrieved from the National Center for Biotechnology Information (NCBI) Genome Database (accessed in October 2022). We also referenced the genome information of *E. akaara* reported by Ge et al., 2019.

### 2.3 Transcriptome assembly and inference of ortholog relationships

For the five species with public reference genomes, the number of transcript sequences obtained using StringTie (Pertea et al., 2015) with the “‐‐merge” option is shown in Table 2. For black sea bass, (without a reference genome), the number of contiguous sequences assembled using Trinity (Grabherr et al., 2011) was 594,726. Among these, 95,941 transcript sequences corresponding to the predicted coding sequences (CDS) were specified as references for expression quantification using Salmon (Patro et al., 2017). The number of protein sequences predicted using TransDecoder is shown in Table 2. Orthology inference and consolidation of orthologs within the three‐spot wrasse identified 7289 orthologs that were common to all species used in this study (Table 2), and thus were considered as candidate sex‐change genes.

### 2.4 Identification of differentially expressed genes

**FIGURE 2:**
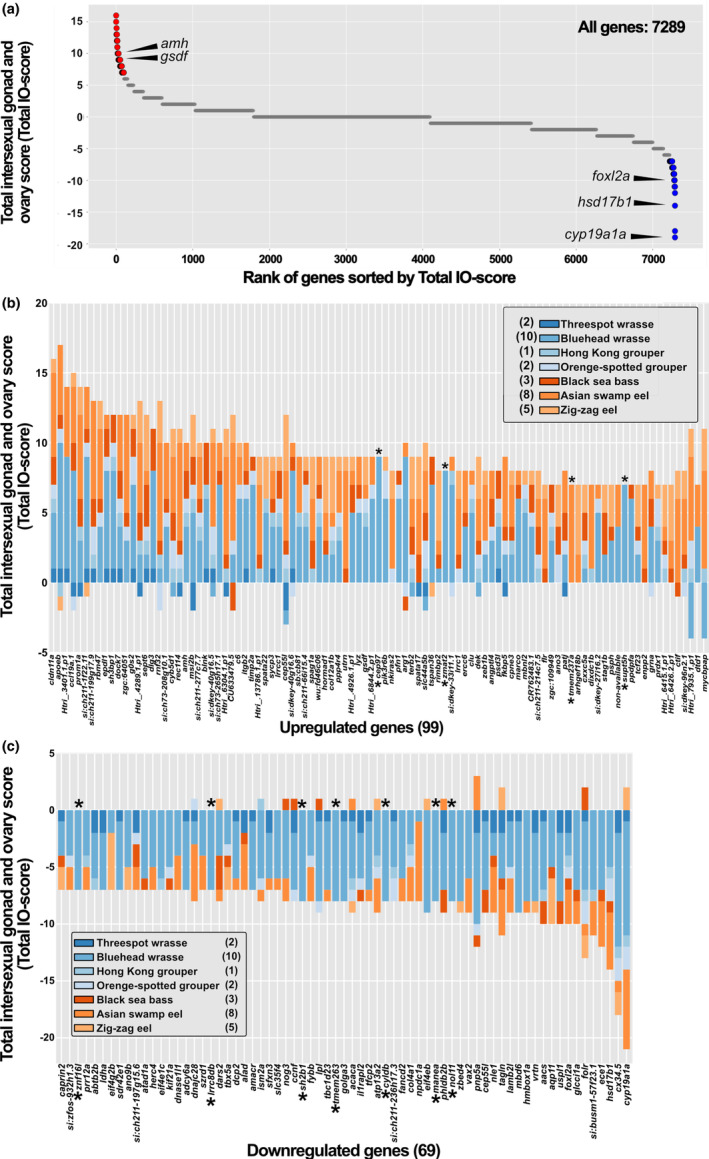
Scatter plot of the total intersexual gonad and ovary (IO)‐score and composition of total IO‐scores for up‐ and downregulated genes. (a) Scatterplot of total IO‐scores for the 7289 genes in descending order. Red circles indicate 99 upregulated genes; blue circles indicate 69 downregulated genes. Gray circles (which have the appearance of lines because they overlap each other) indicate unchanged genes (6 ≤ total IO‐score ≤ −6). Composition of total IO‐scores for up‐ (b, *n* = 99) and downregulated genes (c, *n* = 69). Numbers in parentheses next to species name indicate the number of RNA‐Seq data pairs for each species used in this study. Asterisk indicates a gene for which the total IO‐score is derived from a single species.

The total intersexual gonad and ovary score (IO‐score) was calculated for these 7289 genes (Figure 2a, Table S2, IO‐score calculation; see Methods). Among these, 99 genes were upregulated (Figure 2b, Table S3) and 69 genes were downregulated (Figure 2c, Table S4). Whereas the total IO‐score of these genes was occasionally derived from a single species (up: *cep97*, *zmat2*, *tmem237a*, *supt5h*; down: *znf16l*, *lrrc8db*, *sh2b1*, *tmem263*, *cyldb*, *manea*, *nol11*), in most cases, the total IO‐scores were based on contributions from two or more species. Notably, the upregulated genes included *amh* and *gsdf*, which have been previously reported to play important roles in gonadal sex change, particularly in testicular formation and development, in some protogynous fish. Conversely, the downregulated genes included *cyp19a1a*, *hsd17b1*, and *foxl2a*, which are crucial for the onset of sex change (Table 3).

**TABLE 3:** List of genes that contributed to GO term enrichment.

Up/down	GO ID	Category	GO term	Genes^a^
Up	GO:0007129	Biological process	Homologous chromosome pairing at meiosis	*hormad1, terb2, si:ch211‐199 g17.9*
Up	GO:0007369	Biological process	Gastrulation	*zeb1b, tmem237a, col12a1b, pfn1, enpp2*
Up	GO:1902531	Biological process	Regulation of intracellular signal transduction	*cep55l, psd3l, arhgef18b, ccl19a.1, sh3bp1, enpp2, pik3r6b*
Up	GO:0005929	Cellular component	Cilium	*septin6, prom1a, tmem237a, si:ch211‐66i15.4, flr*
Up	GO:0007281	Biological process	Germ cell development	*hormad1, gsdf, sycp3*
Down	GO:0010817	Biological process	Regulation of hormone levels	*cyp19a1a, aacs, hsd17b1, ece1*
Down	GO:0006413	Biological process	Translational initiation	*eif4g2b, eif4eb, eif4e1c*
Down	GO:0019752	Biological process	Carboxylic acid metabolic process	*lpl, ldha, aacs, amacr, acaca, dars2*
Down	GO:0008202	Biological process	Steroid metabolic process	*hsd17b1, amacr, sdr42e1*
Down	GO:1904888	Biological process	Cranial skeletal system development	*nog3, cep55l, cyldb, ece1*
Down	GO:1901362	Biological process	Organic cyclic compound biosynthetic process	*tbx5a, hsd17b1, alad, acaca, sdr42e1, adcy6a*

^a^ Gene symbols follow the zebrafish gene nomenclature in Ensembl.

### 2.5 Enrichment analysis of differentially expressed genes

**FIGURE 3:**
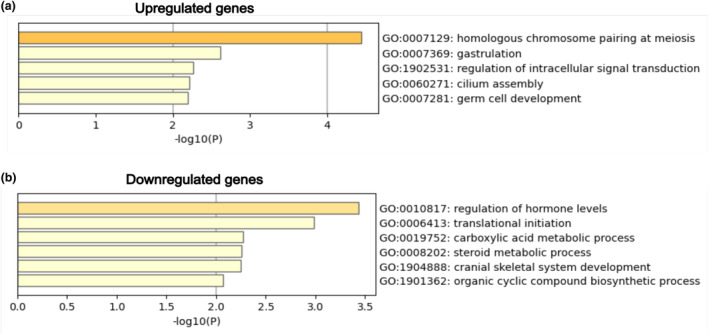
Results of enrichment analysis for differentially expressed genes using Metascape. (a) 99 upregulated genes. (b) 69 downregulated genes.

Sequence homology search of the three‐spot wrasse proteins using ggsearch36 (Pearson & Lipman, 1988) against the reference zebrafish proteins (Ensembl Release 108) (Cunningham et al., 2022) resulted in the identification of 90 homologs among the 99 upregulated genes (Table S3). No corresponding zebrafish orthologs were found for the remaining nine genes that were common to all species analyzed in this study. The results of enrichment analysis using the 90 genes that could be converted to zebrafish orthologs are shown in Figure 3a. The Gene Ontology (GO) terms “homologous chromosome pairing at meiosis” and “germ cell development,” which are related to reproduction, were significantly enriched among the upregulated genes. The specific genes annotated with these GO terms included *hormad1*, *terb2*, *si:ch211‐199 g17.9*, *gsdf*, and *sycp3* (Table 3).

We successfully obtained the corresponding zebrafish homologs for all of the 69 downregulated genes. The results of enrichment analysis are shown in Figure 3b. Among the downregulated genes, GO terms related to the regulation of steroid hormone levels, including “regulation of hormone levels” and “steroid metabolic process,” were significantly enriched. Specific genes annotated with these enriched GO terms included *cyp19a1a*, *aacs*, *hsd17b1*, *ece1*, *amacr*, and *sdr42e1* (Table 3).

### 2.6 Pathways mapping of candidate sex‐change genes

**FIGURE 4:**
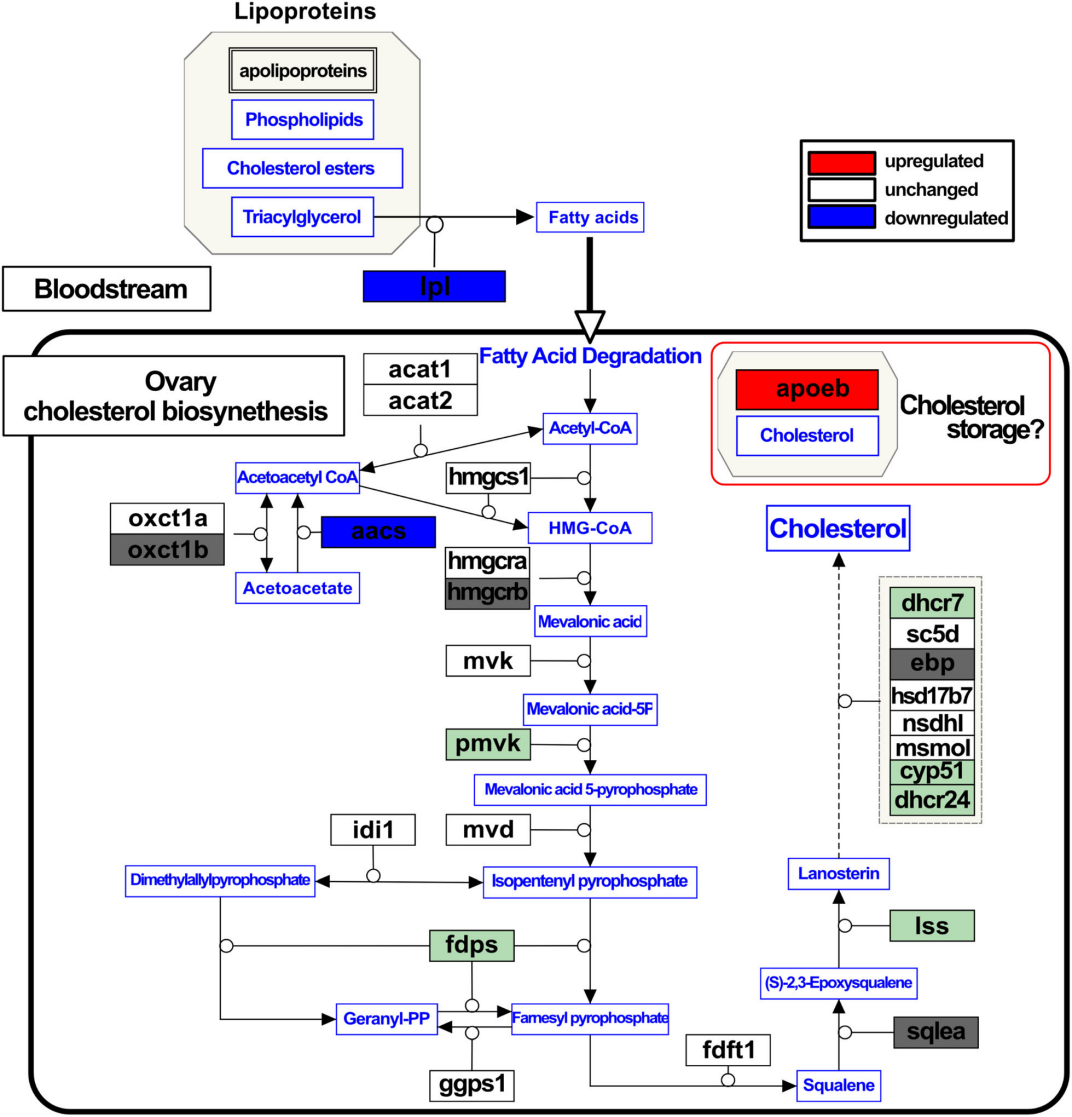
Hypothetical ovarian cholesterol biosynthesis pathway in protogynous fishes. The pathway was visualized using PathVisio (https://pathvisio.org), referencing several fish cholesterol synthesis pathways. Gene products are represented in black text, while metabolites are in blue text. Gene product annotations follow Ensembl Zebrafish gene symbols. Red‐filled boxes represent gene products with confirmed upregulated expression in this study, while blue‐filled boxes denote gene products with downregulated expression. Open boxes represent gene products with no expression variation. Light green‐filled boxes represent gene products not included among the 7289 genes but confirmed to be present in at least the three‐spot wrasse transcriptome. Dark gray‐filled boxes indicate gene products that could not be identified within the three‐spot wrasse transcriptome. Circles extending from gene products represent catalytic reactions, while black arrows indicate the conversion of metabolites. The dashed arrows indicate indirect pathways summarizing multi‐step processes.

Visualization of cholesterol and steroid hormone biosynthesis pathways was conducted based on the GO terms obtained from the enrichment analysis. By referencing pathway information for teleost fishes, pathway diagrams were constructed and loaded with 7289 genes converted to Ensembl zebrafish gene IDs (see Methods). The results showed the mapping of most enzymes involved in the pathway from acetyl‐CoA to cholesterol synthesis (Figure 4). Similarly, when mapping the steroid hormone synthesis pathway, it was confirmed that enzymes involved in the synthesis pathway from cholesterol to estradiol were included among the candidate sex‐change genes (Figure S2).

## 3 DISCUSSION

In this study, we conducted a meta‐analysis of gonadal RNA‐Seq data from seven protogynous fish species using a unified analysis pipeline and threshold. The aim was to compare the transcriptomes of ovaries and sexual transition gonads, with the goal of identifying common genes involved in protogynous gonadal sex change. Toward this end, we first assembled the de novo genome of the three‐spot wrasse. The assembly statistics of the three‐spot wrasse (Table 1) were comparable to those of other Labridae species, such as humphead wrasse (*Cheilinus undulatus*), ballan wrasse (*Labrus bergylta*), and New Zealand spotty (*Notolabrus celidotus*), previously deposited in the NCBI RefSeq database. These results indicated that the de novo genome assembly of the three‐spot wrasse exhibited high quality, enabling us to perform expression analysis and orthology inference with confidence.

In our meta‐analysis, we broadly examined the gonadal sex change process in protogynous fish, focusing on the replacement of ovarian tissue with testicular tissue. At a more detailed level, histological characteristics during the sex change process vary among species. In Labriformes, the early stages of sex change are predominantly characterized by ovarian tissue regression (Nozu et al., 2009; Todd et al., 2019). In contrast, in Perciformes and Synbranchiformes, testicular tissue emerges, and both ovarian and testicular tissues are present simultaneously (Liu & de Mitcheson, 2009; Senthilkumaran et al., 2017; Xue et al., 2021). Additionally, the methods used to obtain transitional gonad samples for RNA‐seq also varied in each project (Table S1). These differences are expected to impact gene expression levels. In this context, we consider the differentially expressed genes identified through the meta‐analysis as crucial factors involved in gonadal transformation across species.

Through this meta‐analysis, we successfully identified sex‐related genes including *cyp19a1a*, *foxl2a*, *hsd17b1*, *amh*, and *gsdf*, that had been previously reported to play a role in sex change in various protogynous fish species (Fan et al., 2022; Horiguchi et al., 2013; Thomas et al., 2019; Todd et al., 2019). Furthermore, our enrichment analysis of downregulated genes highlighted a term related to the “regulation of steroid hormone production.” This finding connects to those of previous reports, which indicate that a rapid decrease in estrogen levels is crucial for female‐to‐male sex change (Kroon & Liley, 2000; Nakamura et al., 1989; Nozu et al., 2009). Thus, by pooling data across species, we were able to identify common genes involved in gonadal sex change. Notably, our meta‐analysis demonstrates the potential of utilizing transcriptome data from multiple experiments to gain insights regarding the mechanisms underlying sex change in fish.

On the other hand, some sex‐related genes, generally identified as related to sex change, showed no differential expression in this study, such as *figla*, *dmrt1*, *csl2*, and *gal3*. Particularly, *dmrt1*, *csl2*, and *gal3* were not included in the set of 7289 orthologs common to all seven species (Table S2). Among these genes, *dmrt1* is known as a key gene in male differentiation (Herpin & Schartl, 2011), and its expression profiles have also been investigated in sex‐changing fish. In some protogynous fish, *dmrt1* expression increases during sex change, coinciding with the appearance of testicular tissue (Nozu et al., 2015; Todd et al., 2019). Since our dataset targets the early stages of sex change in gonads, it is suggested that the expression levels of *dmrt1* were too low for the *dmrt1* transcripts to be assembled.

Our findings demonstrate that *aacs*, annotated with the GO term “regulation of hormone levels,” is downregulated during sex change. Notably, no previous reports have suggested the involvement of *aacs* in sex change, indicating that the encoded protein (acetoacetyl‐CoA synthetase; Aacs) may represent a novel gene associated with this process. Typically, AACS plays a critical role in cholesterol supply. Studies in mice have shown that *Aacs* knockdown decreases total serum cholesterol levels (Hasegawa et al., 2012). Additionally, in rat adrenal glands, inhibition of *AACS* expression through hypermethylation suppresses cholesterol supply and steroid synthesis (Wu et al., 2016). Moreover, apolipoprotein Eb (encoded by *apoeb*, listed among the upregulated genes), has been suggested to participate in cholesterol transportation in zebrafish (Gaudet et al., 2011). Apolipoprotein E, encoded by *APOE* which is the human ortholog of *apoeb*, serves as a crucial lipid transport protein involved in the modulation of cholesterol metabolism and is also synthesized within peripheral tissues (Blue et al., 1983). In addition, a previous study indicates that APOE gene expression exerts suppressive effects on steroid production in adrenal cells (Reyland et al., 1991). Specifically, the high expression of APOE in tissues with active steroid production, including the adrenal glands, appears to shift cholesterol metabolism from delivery to the steroidogenic pathway to storage of esterified cholesterol, resulting in a decrease in steroid production levels (Thorngate et al., 2002). These observations strongly support the hypothesis that the downregulation of *aacs* and the upregulation of *apoeb* during sex change contribute to suppression of the cholesterol supply and entire steroidogenesis.

Cholesterol serves as a precursor for steroid hormones, including estrogens, androgens, and glucocorticoids (Miller, 2008; Payne & Hales, 2004; Pikuleva, 2006), which play crucial roles in protogynous sex change. Previous studies have shown that a decline in endogenous estrogens, particularly E2, triggers the transition from ovary to testis in protogynous fish (Kroon & Liley, 2000; Nakamura et al., 1989; Nozu et al., 2009). E2 is synthesized from testosterone via an aromatase enzyme, encoded by *cyp19a1a*. Downregulation of *cyp19a1a* expression at the onset of sex change leads to reduced E2 production. Studies on *M. albus* and *T. bifasciatum* have revealed higher DNA methylation levels of the *cyp19a1a* gene upstream region in the testes than those in the ovaries, suggesting that epigenetic modifications may contribute to the suppression of *cyp19a1a* expression during gonadal sex change (Todd et al., 2019; Zhang et al., 2013). Our present findings could contribute one more “piece of the puzzle,” suggesting that the trigger of gonadal transformation may be attributed not only to the decreased E2 production by the downregulation of aromatase activity but also to the suppression of the cholesterol supply, the precursor of steroid hormones, leading to the inhibition of the entire steroid biosynthesis.

In fish, it is suggested that synthesized cholesterol affects the production of steroid hormones (Bera et al., 2020; Sharpe et al., 2006). This is also supported by reports that the exposure HMG‐CoA reductase inhibitors in zebrafish leads to a decrease in steroid hormone levels (Al‐Habsi et al., 2016). The cholesterol synthesis pathway (mevalonate pathway) primarily uses acetyl‐CoA as a substrate, and *aacs* might be involved in the supply of this acetyl‐CoA (Figure 4). Additionally, a decrease in the expression of lipoprotein lipase (*lpl*) has been observed (Figure 4, Table S4). Lipoprotein lipase is involved in the biosynthetic process of fatty acid which serves as one of the sources of acetyl‐CoA. This suggests the possibility of a concurrent suppression of fatty acid supply to the gonads. Both results could lead to support the hypothesis that the decrease in steroid hormone production is due to a reduced supply of cholesterol caused by inhibition of cholesterol synthesis. Although this hypothesis requires further investigation and an understanding of cholesterol dynamics during sex change, additional exploration is warranted. Notably, a study on medaka (*Oryzias latipes*) subjected to starvation conditions implied a potential link between masculinization and lower cholesterol levels (Sakae et al., 2020). Specifically, female‐to‐male sex reversal was observed in starved medaka larvae, a gonochoristic fish, together with decreased cholesterol levels in the starved samples. However, the precise pathways underlying masculinization and cholesterol dynamics remain unknown. In general, cholesterol is synthesized not only in the liver and adrenal glands but also in the gonads (Bera et al., 2020; Gwynne & Strauss, 1982; Sharpe et al., 2006). Therefore, gaining a comprehensive understanding of the lipid metabolic mechanisms in fish gonads, including cholesterol synthesis and transportation during sex change, may provide valuable insights regarding the mechanisms underlying gonadal transformation. To clarify this perspective, the remaining challenge lies in providing experimental evidence regarding whether it is possible to control gonadal transformation by actually regulating cholesterol levels within the body.

## 4 CONCLUSIONS

In this study, our objective was to compare the transcriptomes of sex‐transitional gonads with those of ovaries using a unified pipeline to identify common genes involved in gonadal sex change across protogynous fishes. We successfully constructed a high‐quality de novo genome assembly applicable for genome‐wide analysis in the protogynous three‐spot wrasse and acquired newly generated gonadal transcriptome data. Leveraging this information, we conducted a comprehensive meta‐analysis by integrating both public and newly derived gonadal transcriptome data from multiple protogynous fishes spanning three orders. Through this analysis, we identified 168 differentially expressed genes between the two gonadal states, suggesting their potential universal role in gonadal sex change across diverse taxa of protogynous fishes. Notably, we identified novel genes that were not previously associated with gonadal sex change. In particular, we identified cholesterol dynamics as an important candidate factor regulating the sex change process. Moreover, this finding highlights the potential of transcriptomics meta‐analysis as a valuable approach to uncover overlooked mechanisms underlying conserved biological processes including sex change. We anticipate that further data‐driven research utilizing these novel sex change‐related genes will open new horizons in sex change research.

## 5 EXPERIMENTAL PROCEDURES

### 5.1 Animals

The adult female three‐spot wrasse specimens collected in the field by local fisherman in the northern region of Okinawa, Japan were purchased and employed for genome and RNA sequencing. These specimens were subsequently cultivated in 1000 L tanks receiving a continuous supply of unregulated seawater, and were subjected to natural photoperiod conditions until sampling.

The maintenance and handling of fish in this study were conducted in accordance with the “Guidelines for Animal Experiments of the Okinawa Churashima Foundation,” with the same consideration for animal care and welfare as that for higher vertebrates, including reptiles, birds, and mammals. Animal ethical review and approval were not required for the present study because the guidelines stipulated that approval from the Institutional Animal Care and Use Committee of Okinawa Churashima Foundation is required for “higher” vertebrates, and is waived for “lower” vertebrates including fish.

### 5.2 Genome sequencing and assembly for the three‐spot wrasse

Muscle tissue from a mature female individual was used for genome sequencing. The specimen was randomly selected from cultivated tank. After anesthetizing the fish with 0.05% v/v 2‐phenoxyethanol (FUJIFILM Wako Pure Chemical), the muscle tissue was dissected, immediately frozen in liquid nitrogen, and stored at −80°C until genomic DNA extraction. High‐molecular‐weight DNA was extracted from muscle using a NucleoBond AXG column (Macherey–Nagel), followed by purification with phenol–chloroform. The concentration of the extracted DNA was measured using Qubit (Thermo Fisher Scientific Inc., Waltham, MA, USA), and its size distribution was first analyzed with a TapeStation 4200 (Agilent Technologies Inc., Santa Clara, CA, USA) to ensure high integrity, and subsequently analyzed via pulse‐field gel electrophoresis on a CHEF DR‐II system (Bio‐Rad Laboratories, Inc., Hercules, CA, USA) to ensure a size range of 20–100 kb.

To obtain long‐read sequence data, a SMRT sequence library was constructed using the SMRTbell Express Template Prep Kit 2.0 (PacBio, Menlo Park, CA, USA) and sequenced on a PacBio Sequel II system. The sequencing output was processed to generate circular consensus sequences to obtain HiFi sequence reads. Adapter sequences were removed from the HiFi reads using HiFiAdapterFilt (Sim et al., 2022). The obtained HiFi sequence reads were assembled using the hifiasm (v0.16.1) assembler (Cheng et al., 2021) with default parameters. The obtained contigs were first scaffolded with the P_RNA_scaffolder (Zhu et al., 2018) and further scaffolded using Hi‐C data as follows. The Hi‐C library was prepared with the in situ method using the muscle tissue of the individual used for DNA extraction according to the iconHi‐C protocol (Kadota et al., 2020), employing the restriction enzymes DpnII and HinfI, and sequenced on a HiSeq X sequencing platform (Illumina, Inc., San Diego, CA, USA). The obtained Hi‐C read pairs, amounting to 232 million read pairs, were aligned to the HiFi sequence contigs using the Juicer program (Durand et al., 2016), and the HiFi sequence contigs were scaffolded via the 3d‐dna (version 201008) to be consistent with the chromatin contact profiles through manual curation on JuiceBox v1.11.08 (Dudchenko et al., 2018). The continuity and completeness of the resulting genome assembly were assessed using the webserver gVolante (Nishimura et al., 2017), in which the pipeline BUSCO (v5.4, reference datasets: vertebrata_odb10) was implemented (Manni et al., 2021).

### 5.3 RNA‐Seq of gonadal tissue in the three‐spot wrasse

Based on previous studies (Horiguchi et al., 2013; Nozu et al., 2009), we triggered a sex change in three‐spot wrasse *n* = 5 females (body length; 8.0–9.3 cm, body weight; 12.4–20.9 g) to obtain transitional gonads through treatment with an aromatase inhibitor (AI), which exerts inhibitory effects on the enzyme responsible for estrogen synthesis. The collected gonads were cut into small pieces, one piece was immersed in TRIzol reagent (Invitrogen) and the rest was fixed in 4% paraformaldehyde/1× phosphate‐buffered saline (PBS) for histological observation. Total RNA in each sample was extracted promptly in accordance with the TRIzol instruction manual. The extracted total RNA was dissolved in RNase‐free water and stored at −80°C until RNA‐Seq analysis. We selected two treated and two control samples for RNA‐Seq analysis on the basis of histological observation (Figure S3).

Beads with oligo (dT) were used to isolate poly(A) mRNA after the total RNA was collected from the samples. Fragmentation buffer was added to disrupt mRNA into short fragments. Using these short fragments as templates, a random hexamer primer was used to synthesize the first‐strand cDNA. Second‐strand cDNA was synthesized using buffer, dNTPs, Rhase H, and DNA polymerase I. Short fragments were purified using the QiaQuick PCR extraction kit (Qiagen) and resolved with EB buffer for end reparation and adding an “A” base. Subsequently, the short fragments were connected to sequencing adapters. Following agarose gel electrophoresis, suitable fragments were selected as templates for PCR amplification. Finally, the library was sequenced using an Illumina HiSeq 2000 as 2 × 90 bp paired‐ends. The acquired reads were subjected to the quality control procedures described below and used for downstream expression analysis.

### 5.4 Retrieval and acquisition of RNA‐Seq data in protogynous fishes from public resources

To obtain RNA‐Seq data from the gonads of protogynous fishes in public databases, we accessed SRA (Kodama et al., 2011) (accessed in October 2022) to search SRA data containing the following keywords: “bony fishes (AND ovary OR gonad).” SRA data were converted into FASTQ files using the Fasterq‐dump program of the SRA Toolkit (v3.0.0).

### 5.5 Quality control of RNA‐Seq data

To ensure the integrity of the RNA‐Seq reads, trimming and quality control procedures were performed. Specifically, we used Trim Galore (v0.6.7) with the option “‐‐quality 20 ‐‐paired” to eliminate low‐quality bases from paired‐end reads (Trim Galore, 2022).

### 5.6 Read mapping against the reference genome

For species with available genomic information, RNA‐Seq reads were mapped to the reference genome sequences using HISAT2 (v2.2.1) (Kim et al., 2019) with the parameter “‐q ‐dta ‐x”. The resulting SAM files were sorted and converted into BAM files using SAMtools software (v1.15.1) (Danecek et al., 2021).

### 5.7 Generating General Transfer Format (gtf) files based on genome sequence and RNA‐Seq reads for reference gene annotation when quantifying expression values

To ensure consistency in the use of annotation information for expression quantification in species with available reference genomes, we employed the merge function of StringTie (v2.1.7) (Pertea et al., 2015) to generate a gtf file that served as a guide for annotations. The transcriptome assembly for each fish was conducted using StringTie with default settings based on the mapping results (BAM file). The resulting gtf files were then merged using StringTie with the “‐‐merge” option, producing a unified file as the “merged.gtf.” This “merged.gtf” file contained a non‐redundant set of assembled transcripts for each species. Generated “merged.gtf” files in each species were designated as the reference annotation files for quantifying transcript expression.

### 5.8 Quantification of transcripts expression and detection of differentially expressed transcripts

When reanalyzing high‐throughput data from multiple experiments, it is crucial to consider batch effects (Leek et al., 2010). In the present study, we, therefore, calculated the transcript per million (TPM) value for each transcript and utilized the difference in TPM values between the paired data as an indicator for identifying differentially expressed transcripts.

Prior to expression quantification, we performed a principal component analysis (PCA) using count data from mapped reads to examine the expression profiles of ovaries and gonads in each species (Figure S4). The PCA and visualization were conducted using the “pcaExplorer” package (version 2.30.0) (Marini & Binder, 2019) in the R program.

Transcript expression was quantified in species with available genome annotation using StringTie, specifying the parameters as “‐G merged.gtf ‐e”. The resulting gtf file for each sample provided the TPM values, which were used for downstream analysis.

TPM values were computed for species lacking reference genomes using Salmon (v.1.9.0) (Patro et al., 2017) with the parameters “quant ‐I index ‐l A”. To generate the transcriptome sequences required for Salmon, we performed transcriptome assembly using the Trinity (v2.13.1) assembler (Grabherr et al., 2011) with default settings. Open reading frames (ORFs) and CDS were predicted from the transcriptome assembly using TransDecoder (v5.5.0) (Haas, 2018) with the same settings as described below. The resulting gff3 file obtained from the TransDecoder run was used to extract transcript sequences corresponding to the predicted CDS from the transcriptome assembly. The extracted sequence set was then utilized as the reference transcriptome after eliminating duplicate sequences using seqkit (v2.3.0) (Zou et al., 2016) with the “rmdup ‐s” command.

To assess changes in transcript expression, the expression ratio was determined using TPM values obtained from intersexual gonad and ovary transcriptomes (referred to as the IO‐ratio). The IO‐ratio for each transcript was calculated using Equation (1).

(1) $$\;\mathrm{IO}\;\text{ratio}=\log_{2}\left({\mathrm{TPM}}_{\text{intersexual}}+0.01\right)–\log_{2}\left({\mathrm{TPM}}_{\text{ovary}}+0.01\right),$$

This calculation was performed for all pairs of intersexual gonad and ovary transcriptomes. To convert zeros to logarithms, a value of “0.01” was added to the TPM of each transcript. Transcripts with an IO‐ratio > log_2_(5) (indicating a 5‐fold expression change) were classified as “upregulated,” whereas transcripts with an IO‐ratio < −log_2_(5) (representing a 0.2‐fold expression change) were considered “downregulated.” Transcripts outside these thresholds were deemed “unchanged.” The threshold was set based on the ±2 SD (standard deviation) range of expression ratios of transcripts in each species (Table S5). To determine differential expression between the intersexual gonad and ovary, an IO‐score was calculated for each transcript by subtracting the count of “downregulated” pairs from that of “upregulated” pairs, as shown in Equation (2).

(2) $$\mathrm{IO}\;\text{score}=\left({\text{count number}}_{\text{upregulated}}\right)–\left({\text{count number}}_{\text{downregulated}}\right),$$

Using orthology inference as described below, the total IO‐score for each transcript was calculated by summing the IO‐scores of all species. Total IO‐score thresholds of 7 and −7 were employed to identify transcripts that were differentially upregulated and downregulated, respectively. The thresholds were arbitrarily set based on 20% above the maximum or below the minimum values (31 or −31). All calculations for IO‐ratios and IO‐scores were performed using R (v 4.2.1) (R Core Team, 2022).

### 5.9 Orthology inference among species

GFFread (v0.12.1) (Pertea & Pertea, 2020) was utilized along with the generated “merged.gtf” file to extract transcript sequences from the reference genomes. For species without reference genomes, transcript sequences were obtained via assembly using Trinity, as described previously. TransDecoder was used to identify the coding regions and translate them into amino acid sequences for all species. ORFs were extracted from the transcriptome sequences using TransDecoder Longorfs with the parameters “‐m 100.” CDS were then predicted using TransDecoder Predict with default settings, and the protein sequences converted from the predicted CDS were acquired. Subsequently, a reciprocal homology search was conducted between the predicted protein sequences of the three‐spot wrasse and those of other species, and the reciprocal best hit (RBH) was identified as the ortholog. The program Diamond (v2.0.15) (Buchfink et al., 2014) was used for the RBH search using the parameters “‐‐ultra‐sensitive ‐e 1e‐3”. To extract the orthologs shared among all species, we consolidated the RBH results with the three‐spot wrasse‐predicted protein IDs.

### 5.10 Gene set enrichment analysis

Differentially expressed gene sets were analyzed using the web tool Metascape (v3.5) (Zhou et al., 2019), which enables gene set enrichment analysis. However, Metascape only supports the analysis of zebrafish among teleosts. Therefore, prior to analysis, the predicted protein sequences of the three‐spot wrasse were mapped to the corresponding zebrafish Ensembl protein IDs. To obtain orthologous information, we utilized the ggsearch36 program from the FASTA package (Pearson & Lipman, 1988) to globally align the predicted protein sequences of the three‐spot wrasse with the proteome sequences of zebrafish (Ensembl Release 108) (Cunningham et al., 2022).

### 5.11 Visualization of cholesterol and steroid hormone biosynthesis pathways and orthologs mapping to the pathways

The hypothetical cholesterol biosynthesis and steroid hormone biosynthesis pathway in teleost fish was illustrated using PathVisio (Murphy et al., 2015). Modifying information from WikiPathways (Pico et al., 2008) intended for zebrafish (WP1387, https://www.wikipathways.org/instance/WP1387) and data from the yellow perch, *Perca flavescens* (Kemski et al., 2020), facilitated the depiction of the cholesterol biosynthesis pathway. Additionally, the steroid hormone biosynthesis pathway was constructed by adjusting information sourced from various fish models (Craft et al., 2015; Fernandino et al., 2013; Li et al., 2006; Liu et al., 2022; Nagahama et al., 2021). Moreover, through PathVisio, 7289 candidate sex‐change genes, converted into Ensembl zebrafish gene IDs, were mapped onto the established biosynthesis pathways.

## AUTHOR CONTRIBUTIONS

R.N. and H.B. conceived and designed the study. M.K. and S.K. performed genome sequence and de novo genome assembly of three‐spot wrasse. R.N. and M.N. RNA‐Seq data production of three‐spot wrasse. R.N. performed RNA‐seq data analysis and orthology inference. R.N., M.N., S.K., and H.B. wrote a draft manuscript. All authors read and approved the final manuscript.

## CONFLICT OF INTEREST STATEMENT

The authors declare no conflicts of interest.

## Supporting information


**Figure S1.** Schematic diagram of the analysis pipeline in this study. The consistent line style of the arrows indicates the utilization of common tools for conducting data processing. The letters in parentheses next to the species names represent the following order names: L, Labriformes; P, Perciformes; S, Synbranchiformes.
**Figure S2.** Hypothetical ovarian steroid hormones biosynthesis pathway in protogynous fishes. The pathway was visualized using PathVisio (https://pathvisio.org), referencing several fish cholesterol synthesis pathways. Gene products are represented in black text, while metabolites are in blue text. Gene product annotations follow Ensembl Zebrafish gene symbols. Red‐filled boxes represent gene products with confirmed upregulated expression in this study, while blue‐filled boxes denote gene products with downregulated expression. Open boxes represent gene products with no expression variation. Light green‐filled boxes represent gene products not included among the 7289 factors but confirmed to be present in at least the three‐spot wrasse transcriptome. Dark gray‐filled boxes indicate gene products that could not be identified within the three‐spot wrasse transcriptome. Circles extending from gene products represent catalytic reactions, while black arrows indicate the conversion of metabolites.
**Figure S3.** Histological sections of control ovaries and transitional gonads treated AI for 7 days in the threespot wrasse, *Halichoeres trimaculatus*.
**Figure S4.** Principal component analysis (PCA) results of gene expression data from each species used in this study. The status of transitional gonads is indicated according to the metadata descriptions in the Supplementary Table 1. The black line circles in *Monopterus albus* represent data derived from different bioprojects. The analysis and visualization were performed using the R program (version 4.4.0) with the ‘pcaExplorer’ package (version 2.30.0; Marini & Binder, 2019).

**Table S1.** Metadata of all RNA‐seq data used in this study.
**Table S2.** Details of IO‐score in each gene (7289) and fish.
**Table S3.** Orthology information of zebrafish protein corresponding to 99 up‐regulated factors.
**Table S4.** Orthology information of zebrafish protein corresponding to 69 down‐regulated factors.
**Table S5.** Statistics on expression ratios of common orthologs in each species.

## Data Availability

All sequencing data, including assembled sequences and raw sequence reads, were deposited in the DNA Data Bank of Japan (DDBJ) under the umbrella BioProject accession number PRJDB15835. The genome assembly was deposited in the DDBJ under accession numbers BSYP01000001–BSYP01000227. Additionally, raw sequence reads were deposited in the DDBJ under the following accession numbers: DRR466686 (PacBio HiFi reads), DRR486910 (Illumina HiC reads), and DRR483512–DRR483515 (Illumina RNA‐Seq reads).

## References

[gtc13166-bib-0001] Al‐Habsi, A. A. , Massarsky, A. , & Moon, T. W. (2016). Exposure to gemfibrozil and atorvastatin affects cholesterol metabolism and steroid production in zebrafish (*Danio rerio*). Comparative Biochemistry and Physiology Part B: Biochemistry and Molecular Biology, 199, 87–96. 10.1016/j.cbpb.2015.11.009 26627126

[gtc13166-bib-0002] Atz, J. W. (1964). Intersexuality in fishes. In C. N. Armstrong & A. J. Marshall (Eds.), Intersexuality in vertebrates including man (pp. 145–232). Academic press.

[gtc13166-bib-0003] Bera, A. , Chadha, N. K. , Dasgupta, S. , Chakravarty, S. , & Sawant, P. B. (2020). Hypoxia‐mediated inhibition of cholesterol synthesis leads to disruption of nocturnal sex steroidogenesis in the gonad of koi carp, *Cyprinus carpio* . Fish Physiology and Biochemistry, 46, 2421–2435. 10.1007/s10695-020-00887-5 33034795

[gtc13166-bib-0004] Bhandari, R. K. , Komuro, H. , Nakamura, S. , Higa, M. , & Nakamura, M. (2003). Gonadal restructuring and correlative steroid hormone profiles during natural sex change in protogynous honeycomb grouper (*Epinephelus merra*). Zoological Science, 20, 1399–1404. 14624040 10.2108/zsj.20.1399

[gtc13166-bib-0005] Blue, M. L. , Williams, D. L. , Zucker, S. , Khan, S. A. , & Blum, C. B. (1983). Apolipoprotein E synthesis in human kidney, adrenal gland, and liver. Proceedings of the National Academy of Sciences of the United States of America, 80, 283–287. 10.1073/pnas.80.1.283 6572003 PMC393357

[gtc13166-bib-0006] Bono, H. (2021). Meta‐analysis of oxidative transcriptomes in insects. Antioxidants, 10, 345. 10.3390/antiox10030345 33669076 PMC7996572

[gtc13166-bib-0007] Breton, T. S. , Kenter, L. W. , Greenlaw, K. , Montgomery, J. , Goetz, G. W. , Berlinsky, D. L. , & Luckenbach, J. A. (2019). Initiation of sex change and gonadal gene expression in black sea bass (*Centropristis striata*) exposed to exemestane, an aromatase inhibitor. Comparative Biochemistry and Physiology Part A: Molecular & Integrative Physiology, 228, 51–61. 10.1016/j.cbpa.2018.10.024 PMC6311140 30414915

[gtc13166-bib-0008] Buchfink, B. , Xie, C. , & Huson, D. H. (2014). Fast and sensitive protein alignment using DIAMOND. Nature Methods, 12, 59–60. 10.1038/nmeth.3176 25402007

[gtc13166-bib-0009] Cheng, H. , Concepcion, G. T. , Feng, X. , Zhang, H. , & Li, H. (2021). Haplotype‐resolved de novo assembly using phased assembly graphs with Hifiasm. Nature Methods, 18, 170–175. 10.1038/s41592-020-01056-5 33526886 PMC7961889

[gtc13166-bib-0010] Craft, J. A. , Bain, P. A. , Papanicolaou, A. , & Kumar, A. (2015). Identification of putative nuclear receptors and steroidogenic enzymes in Murray‐Darling rainbowfish (*Melanotaenia fluviatilis*) using RNA‐seq and de novo transcriptome assembly. PLoS One, 10, e0142636. 10.1371/journal.pone.0142636 26599404 PMC4658143

[gtc13166-bib-0011] Cunningham, F. , Allen, J. E. , Allen, J. , Alvarez‐Jarreta, J. , Amode, M. R. , Armean, I. M. , Austine‐Orimoloye, O., Azov, A. G., Barnes, I., Bennett, R., Berry, A., Bhai, J., Bignell, A., Billis, K., Boddu, S., Brooks, L., Charkhchi, M., Cummins, C., Da Rin Fioretto, L., … Flicek, P. (2022). Ensembl 2022. Nucleic Acids Research, 50, D988–D995. 10.1093/nar/gkab1049 34791404 PMC8728283

[gtc13166-bib-0012] Danecek, P. , Bonfield, J. K. , Liddle, J. , Marshall, J. , Ohan, V. , Pollard, M. O. , Whitwham, A., Keane T., McCarthy, S. A., Davies, R. M., & Li, H. (2021). Twelve years of SAMtools and BCFtools. GigaScience, 10, giab008. 10.1093/gigascience/giab008 33590861 PMC7931819

[gtc13166-bib-0013] Dudchenko, O. , Shamim, M. S. , Batra, S. S. , Durand, N. C. , Musial, N. T. , Mostofa, R. , Pham, M., Hilaire, B. G. S., Yao, W., Stamenova, E., Hoeger, M., Nyquist, S. K., korchina, V., Pletch, K., Flanagan, J. P., Tomaszewicz, A., McAloose, D., Estrada, C. P., Novak, B. J., … Aiden, E. L. (2018). The Juicebox assembly tools module facilitates de novo assembly of mammalian genomes with chromosome‐length scaffolds for under $1000. bioRxiv, 254797. 10.1101/254797

[gtc13166-bib-0014] Durand, N. C. , Shamim, M. S. , Machol, I. , Rao, S. S. P. , Huntley, M. H. , Lander, E. S. , & Aiden, E. L. (2016). Juicer provides a one‐click system for analyzing loop‐resolution hi‐C experiments. Cell Systems, 3, 95–98. 10.1016/j.cels.2016.07.002 27467249 PMC5846465

[gtc13166-bib-0015] Fan, M. , Yang, W. , Zhang, W. , & Zhang, L. (2022). The ontogenic gonadal transcriptomes provide insights into sex change in the ricefield eel *Monopterus albus* . BMC Zoology, 7, 56. 10.1186/s40850-022-00155-4 37170354 PMC10127409

[gtc13166-bib-0016] Fernandino, J. I. , Hattori, R. S. , Moreno Acosta, O. D. , Strussmann, C. A. , & Somoza, G. M. (2013). Environmental stress‐induced testis differentiation: Androgen as a by‐product of cortisol inactivation. General and Comparative Endocrinology, 192, 36–44. 10.1016/j.ygcen.2013.05.024 23770022

[gtc13166-bib-0017] Gaudet, P. , Livstone, M. S. , Lewis, S. E. , & Thomas, P. D. (2011). Phylogenetic‐based propagation of functional annotations within the gene ontology consortium. Briefings in Bioinformatics, 12, 449–462. 10.1093/bib/bbr042 21873635 PMC3178059

[gtc13166-bib-0018] Ge, H. , Lin, K. , Shen, M. , Wu, S. , Wang, Y. , Zhang, Z. , Wang, Z., Zhang, Y., Huang, Z., Zhou, C., Lin, Q., Wu, J., Liu, L., Hu, J., Huang, Z., & Zheng, L. (2019). De novo assembly of a chromosome‐level reference genome of red‐spotted grouper (Epinephelus akaara) using nanopore sequencing and hi‐C. Molecular Ecology Resources, 19, 1461–1469. 10.1111/1755-0998.13064 31325912 PMC6899872

[gtc13166-bib-0019] Grabherr, M. G. , Haas, B. J. , Yassour, M. , Levin, J. Z. , Thompson, D. A. , Amit, I. , Adiconis, X., Fan, L., Raychowdhury, R., Zeng, Q., Chen, Z., Mauceli, E., Hacohen, N., Gnirke, A., Rhind, N., di Palma, F., Birren, B. W., Nusbaum, C., Lindblad‐Toh, K., … Regev, A. (2011). Full‐length transcriptome assembly from RNA‐seq data without a reference genome. Nature Biotechnology, 29, 644–652. 10.1038/nbt.1883 PMC3571712 21572440

[gtc13166-bib-0020] Gwynne, J. T. , & Strauss, J. F. (1982). The role of lipoproteins in steroidogenesis and cholesterol metabolism in steroidogenic glands. Endocrine Reviews, 3, 299–329. 10.1210/edrv-3-3-299 6288367

[gtc13166-bib-0021] Haas, B. (2018). TransDecoder (v5.5.0); October 2022. https://github.com/TransDecoder/TransDecoder.

[gtc13166-bib-0022] Hasegawa, S. , Noda, K. , Maeda, A. , Matsuoka, M. , Yamasaki, M. , & Fukui, T. (2012). Acetoacetyl‐CoA synthetase, a ketone body‐utilizing enzyme, is controlled by SREBP‐2 and affects serum cholesterol levels. Molecular Genetics and Metabolism, 107, 553–560. 10.1016/j.ymgme.2012.08.017 22985732

[gtc13166-bib-0023] He, Z. , Deng, F. , Yang, D. , He, Z. , Hu, J. , Ma, Z. , Zhang, Q., He, J., Ye, L., Chen, H., He, L., Luo, J., Xiong, S., Luo, W., Yang, S., Gu, X., & Yan, T. (2022). Crosstalk between sex‐related genes and apoptosis signaling reveals molecular insights into sex change in a protogynous hermaphroditic teleost fish, ricefield eel *Monopterus albus* . Aquaculture, 552, 737918. 10.1016/j.aquaculture.2022.737918

[gtc13166-bib-0024] Herpin, A. , & Schartl, M. (2011). Dmrt1 genes at the crossroads: A widespread and central class of sexual development factors in fish. FEBS Journal, 278, 1010–1019. 10.1111/j.1742-4658.2011.08030.x 21281449

[gtc13166-bib-0025] Horiguchi, R. , Nozu, R. , Hirai, T. , Kobayashi, Y. , Nagahama, Y. , & Nakamura, M. (2013). Characterization of gonadal soma‐derived factor expression during sex change in the protogynous wrasse, *Halichoeres trimaculatus* . Developmental Dynamics, 242, 388–399. 10.1002/dvdy.23929 23335393

[gtc13166-bib-0026] Horiguchi, R. , Nozu, R. , Hirai, T. , Kobayashi, Y. , & Nakamura, M. (2018). Expression patterns of sex differentiation‐related genes during gonadal sex change in the protogynous wrasse, *Halichoeres trimaculatus* . General and Comparative Endocrinology, 257, 67–73. 10.1016/j.ygcen.2017.06.017 28663108

[gtc13166-bib-0027] Kadota, M. , Nishimura, O. , Miura, H. , Tanaka, K. , Hiratani, I. , & Kuraku, S. (2020). Multifaceted hi‐C benchmarking: What makes a difference in chromosome‐scale genome scaffolding? GigaScience, 9, giz158. 10.1093/gigascience/giz158 31919520 PMC6952475

[gtc13166-bib-0028] Kemski, M. M. , Rappleye, C. A. , Dabrowski, K. , Bruno, R. S. , & Wick, M. (2020). Transcriptomic response to soybean meal‐based diets as the first formulated feed in juvenile yellow perch (*Perca flavescens*). Scientific Reports, 10, 3998. 10.1038/s41598-020-59691-z 32132548 PMC7055240

[gtc13166-bib-0029] Kim, D. , Paggi, J. M. , Park, C. , Bennett, C. , & Salzberg, S. L. (2019). Graph‐based genome alignment and genotyping with HISAT2 and HISAT‐genotype. Nature Biotechnology, 37, 907–915. 10.1038/s41587-019-0201-4 PMC7605509 31375807

[gtc13166-bib-0030] Kobayashi, T. , Kajiura‐Kobayashi, H. , Guan, G. , & Nagahama, Y. (2008). Sexual dimorphic expression of DMRT1 and Sox9a during gonadal differentiation and hormone‐induced sex reversal in the teleost fish Nile tilapia (*Oreochromis niloticus*). Developmental Dynamics, 237, 297–306. 10.1002/dvdy.21409 18095345

[gtc13166-bib-0031] Kodama, Y. , Shumway, M. , & Leinonen, R. (2011). The sequence read archive: Explosive growth of sequencing data. Nucleic Acids Research, 40, D54–D56. 10.1093/nar/gkr854 22009675 PMC3245110

[gtc13166-bib-0032] Kroon, F. J. , & Liley, N. R. (2000). The role of steroid hormones in protogynous sex change in the blackeye goby, *Coryphopterus nicholsii* (Teleostei: Gobiidae). General and Comparative Endocrinology, 118, 273–283. 10.1006/gcen.2000.7459 10890567

[gtc13166-bib-0033] Kuwamura, T. , Sunobe, T. , Sakai, Y. , Kadota, T. , & Sawada, K. (2020). Hermaphroditism in fishes: An annotated list of species, phylogeny, and mating system. Ichthyological Research, 67, 341–360. 10.1007/s10228-020-00754-6

[gtc13166-bib-0034] Leek, J. T. , Scharpf, R. B. , Bravo, H. C. , Simcha, D. , Langmead, B. , Johnson, W. E. , Geman, D., Baggerly, K., & Irizarry, R. A. (2010). Tackling the widespread and critical impact of batch effects in high‐throughput data. Nature Reviews. Genetics, 11, 733–739. 10.1038/nrg2825 PMC3880143 20838408

[gtc13166-bib-0035] Li, G. L. , Liu, X. C. , Zhang, Y. , & Lin, H. R. (2006). Gonadal development, aromatase activity and P450 aromatase gene expression during sex inversion of protogynous red‐spotted grouper *Epinephelus akaara* (Temminck and Schlegel) after implantation of the aromatase inhibitor, fadrozole. Aquaculture Research, 37, 484–491.

[gtc13166-bib-0036] Liu, M. , & de Mitcheson, Y. S. (2009). Gonad development during sexual differentiation in hatchery‐produced orange‐spotted grouper (*Epinephelus coioides*) and humpback grouper (*Cromileptes altivelis*) (Pisces: Serranidae, Epinephelinae). Aquaculture, 287, 191–202. 10.1016/j.aquaculture.2008.10.027

[gtc13166-bib-0037] Liu, Y. , Kossack, M. E. , McFaul, M. E. , Christensen, L. N. , Siebert, S. , Wyatt, S. R. , Kamei, C. N., Horst, S., Arroyo, N., Drummond, I. A., Juliano, C. E., & Draper, B. W. (2022). Single‐cell transcriptome reveals insights into the development and function of the zebrafish ovary. eLife, 11, e70614. 10.7554/eLife.76014 PMC9191896 35588359

[gtc13166-bib-0038] Lo Nostro, F. , Grier, H. , Andreone, L. , & Guerrero, G. A. (2003). Involvement of the gonadal germinal epithelium during sex reversal and seasonal testicular cycling in the protogynous swamp eel, *Synbranchus marmoratus* Bloch 1795 (Teleostei, Synbranchidae). Journal of Morphology, 257, 107–126. 10.1002/jmor.10105 12740902

[gtc13166-bib-0039] Mank, J. E. , Promislow, D. E. L. , & Avise, J. C. (2006). Evolution of alternative sex‐determining mechanisms in teleost fishes. Biological Journal of the Linnean Society, 87, 83–93. 10.1111/j.1095-8312.2006.00558.x

[gtc13166-bib-0040] Manni, M. , Berkeley, M. R. , Seppey, M. , Simão, F. A. , Zdobnov, E. M. , & Kelley, J. (2021). BUSCO update: Novel and streamlined workflows along with broader and deeper phylogenetic coverage for scoring of eukaryotic, prokaryotic, and viral genomes. Molecular Biology and Evolution, 38, 4647–4654. 10.1093/molbev/msab199 34320186 PMC8476166

[gtc13166-bib-0041] Marini, F. , & Binder, H. (2019). pcaExplorer: An R/Bioconductor package for interacting with RNA‐seq principal components. BMC Bioinformatics, 20, 331. 10.1186/s12859-019-2879-1 31195976 PMC6567655

[gtc13166-bib-0042] Miller, W. L. (2008). Steroidogenic enzymes. In C. E. Flück & W. L. Miller (Eds.), Disorders of the human adrenal cortex (pp. 1–18). S.Karger AG.

[gtc13166-bib-0043] Miyake, Y. , Sakai, Y. , & Kuniyoshi, H. (2012). Molecular cloning and expression profile of sex‐specific genes, Figla and Dmrt1, in the protogynous hermaphroditic fish, *Halichoeres poecilopterus* . Zoological Science, 29, 690–701. 10.2108/zsj.29.690 23030342

[gtc13166-bib-0044] Muncaster, S. , Norberg, B. , & Andersson, E. (2013). Natural sex change in the temperate protogynous Ballan wrasse *Labrus bergylta* . Journal of Fish Biology, 82, 1858–1870. 10.1111/jfb.12113 23731141

[gtc13166-bib-0045] Murphy, R. F. , Kutmon, M. , van Iersel, M. P. , Bohler, A. , Kelder, T. , Nunes, N. , Pico, A. R., & Evelo, C. T. (2015). PathVisio 3: An extendable pathway analysis toolbox. PLoS Computational Biology, 11, e1004085. 10.1371/journal.pcbi.1004085 25706687 PMC4338111

[gtc13166-bib-0046] Nagahama, Y. , Chakraborty, T. , Paul‐Prasanth, B. , Ohta, K. , & Nakamura, M. (2021). Sex determination, gonadal sex differentiation, and plasticity in vertebrate species. Physiological Reviews, 101, 1237–1308. 10.1152/physrev.00044.2019 33180655

[gtc13166-bib-0047] Nakamura, M. , Hourigan, T. F. , Yamauchi, K. , Nagahama, Y. , & Grau, E. G. (1989). Histological and ultrastructural evidence for the role of gonadal steroid hormones in sex change in the protogynous wrasse *Thalassoma duperrey* . Environmental Biology of Fishes, 24, 117–136. 10.1007/bf00001282

[gtc13166-bib-0048] Nishimura, O. , Hara, Y. , Kuraku, S. , & Hancock, J. (2017). gVolante for standardizing completeness assessment of genome and transcriptome assemblies. Bioinformatics, 33, 3635–3637. 10.1093/bioinformatics/btx445 29036533 PMC5870689

[gtc13166-bib-0049] Nozu, R. , Horiguchi, R. , Kobayashi, Y. , & Nakamura, M. (2015). Expression profile of doublesex/male abnormal‐3‐related transcription factor‐1 during gonadal sex change in the protogynous wrasse, *Halichoeres trimaculatus* . Molecular Reproduction and Development, 82, 859–866. 10.1002/mrd.22527 26202688

[gtc13166-bib-0050] Nozu, R. , Kojima, Y. , & Nakamura, M. (2009). Short term treatment with aromatase inhibitor induces sex change in the protogynous wrasse, *Halichoeres trimaculatus* . General and Comparative Endocrinology, 161, 360–364. 10.1016/j.ygcen.2009.01.024 19523378

[gtc13166-bib-0051] Ohta, K. , Hirano, M. , Mine, T. , Mizutani, H. , Yamaguchi, A. , & Matsuyama, M. (2008). Body color change and serum steroid hormone levels throughout the process of sex change in the adult wrasse, *Pseudolabrus sieboldi* . Marine Biology, 153, 843–852. 10.1007/s00227-007-0856-0

[gtc13166-bib-0052] Patro, R. , Duggal, G. , Love, M. I. , Irizarry, R. A. , & Kingsford, C. (2017). Salmon provides fast and bias‐aware quantification of transcript expression. Nature Methods, 14, 417–419. 10.1038/nmeth.4197 28263959 PMC5600148

[gtc13166-bib-0053] Payne, A. H. , & Hales, D. B. (2004). Overview of steroidogenic enzymes in the pathway from cholesterol to active steroid hormones. Endocrine Reviews, 25, 947–970. 10.1210/er.2003-0030 15583024

[gtc13166-bib-0054] Pearson, W. R. , & Lipman, D. J. (1988). Improved tools for biological sequence comparison. Proceedings of the National Academy of Sciences of the United States of America, 85, 2444–2448. 10.1073/pnas.85.8.2444 3162770 PMC280013

[gtc13166-bib-0055] Pertea, G. , & Pertea, M. (2020). GFF utilities: GffRead and GffCompare. F1000Research, 9, 304. 10.12688/f1000research.23297.1 PMC7222033 32489650

[gtc13166-bib-0056] Pertea, M. , Pertea, G. M. , Antonescu, C. M. , Chang, T.‐C. , Mendell, J. T. , & Salzberg, S. L. (2015). StringTie enables improved reconstruction of a transcriptome from RNA‐seq reads. Nature Biotechnology, 33, 290–295. 10.1038/nbt.3122 PMC4643835 25690850

[gtc13166-bib-0057] Pico, A. R. , Kelder, T. , van Iersel, M. P. , Hanspers, K. , Conklin, B. R. , & Evelo, C. (2008). WikiPathways: Pathway editing for the people. PLoS Biology, 6, e184. 10.1371/journal.pbio.0060184 18651794 PMC2475545

[gtc13166-bib-0058] Pikuleva, I. A. (2006). Cholesterol‐metabolizing cytochromes P450. Drug Metabolism and Disposition: The Biological Fate of Chemicals, 34, 513–520. 10.1124/dmd.105.008789 16434543

[gtc13166-bib-0059] R Core Team . (2022). R: A language and environment for statistical computing. https://www.R-project.org/.

[gtc13166-bib-0060] Rabosky, D. L. , Chang, J. , Title, P. O. , Cowman, P. F. , Sallan, L. , Friedman, M. , Kaschner, K., Garilao, C., Near, T. J., Coll, M., & Alfaro, M. E. (2018). An inverse latitudinal gradient in speciation rate for marine fishes. Nature, 559, 392–395. 10.1038/s41586-018-0273-1 29973726

[gtc13166-bib-0061] Rahman, M. R. , Petralia, M. C. , Ciurleo, R. , Bramanti, A. , Fagone, P. , Shahjaman, M. , Wu, L., Sun, Y., Turanli, B., Arga, K. Y., Islam, M. R., Islam, T., & Nicoletti, F. (2020). Comprehensive analysis of RNA‐seq gene expression profiling of brain transcriptomes reveals novel genes, regulators, and pathways in autism spectrum disorder. Brain Sciences, 10, 747. 10.3390/brainsci10100747 PMC7603078 33080834

[gtc13166-bib-0062] Reyland, M. E. , Gwynne, J. T. , Forgez, P. , Prack, M. M. , & Williams, D. L. (1991). Expression of the human apolipoprotein E gene suppresses steroidogenesis in mouse Y1 adrenal cells. Proceedings of the National Academy of Sciences of the United States of America, 88, 2375–2379. 10.1073/pnas.88.6.2375 1848701 PMC51234

[gtc13166-bib-0063] Rodríguez‐Marí, A. , Yan, Y.‐L. , BreMiller, R. A. , Wilson, C. , Cañestro, C. , & Postlethwait, J. H. (2005). Characterization and expression pattern of zebrafish anti‐Müllerian hormone (amh) relative to sox9a, sox9b, and cyp19a1a, during gonad development. Gene Expression Patterns, 5, 655–667. 10.1016/j.modgep.2005.02.008 15939378

[gtc13166-bib-0064] Sakae, Y. , Oikawa, A. , Sugiura, Y. , Mita, M. , Nakamura, S. , Nishimura, T. , Suematsu, M., & Tanaka, M. (2020). Starvation causes female‐to‐male sex reversal through lipid metabolism in the teleost fish, medaka (*Olyzias latipes*). Biology Open, 9, bio050054. 10.1242/bio.050054 32265199 PMC7132775

[gtc13166-bib-0065] Senthilkumaran, B. , Chi, W. , Gao, Y. , Hu, Q. , Guo, W. , & Li, D. (2017). Genome‐wide analysis of brain and gonad transcripts reveals changes of key sex reversal‐related genes expression and signaling pathways in three stages of *Monopterus albus* . PLoS One, 12, e0173974. 10.1371/journal.pone.0173974 28319194 PMC5358790

[gtc13166-bib-0066] Sharpe, R. L. , Drolet, M. , & MacLatchy, D. L. (2006). Investigation of de novo cholesterol synthetic capacity in the gonads of goldfish (*Carassius auratus*) exposed to the phytosterol beta‐sitosterol. Reproductive Biology and Endocrinology, 4, 60. 10.1186/1477-7827-4-60 17118198 PMC1664568

[gtc13166-bib-0067] Shibata, Y. , Paul‐Prasanth, B. , Suzuki, A. , Usami, T. , Nakamoto, M. , Matsuda, M. , & Nagahama, Y. (2010). Expression of gonadal soma derived factor (GSDF) is spatially and temporally correlated with early testicular differentiation in medaka. Gene Expression Patterns, 10, 283–289. 10.1016/j.gep.2010.06.005 20601164

[gtc13166-bib-0068] Sim, S. B. , Corpuz, R. L. , Simmonds, T. J. , & Geib, S. M. (2022). HiFiAdapterFilt, a memory efficient read processing pipeline, prevents occurrence of adapter sequence in PacBio HiFi reads and their negative impacts on genome assembly. BMC Genomics, 23, 157. 10.1186/s12864-022-08375-1 35193521 PMC8864876

[gtc13166-bib-0069] Skaar, K. S. , Nobrega, R. H. , Magaraki, A. , Olsen, L. C. , Schulz, R. W. , & Male, R. (2011). Proteolytically activated, recombinant anti‐mullerian hormone inhibits androgen secretion, proliferation, and differentiation of spermatogonia in adult zebrafish testis organ cultures. Endocrinology, 152, 3527–3540. 10.1210/en.2010-1469 21750047

[gtc13166-bib-0070] Thomas, J. T. , Todd, E. V. , Muncaster, S. , Lokman, P. M. , Damsteegt, E. L. , Liu, H. , Soyano, K., Gleonnec, F., Lamm, M. S., Godwin, J. R., & Gemmell, N. J. (2019). Conservation and diversity in expression of candidate genes regulating socially‐induced female‐male sex change in wrasses. PeerJ, 7, e7032. 10.7717/peerj.7032 31218121 PMC6568253

[gtc13166-bib-0071] Thorngate, F. E. , Strockbine, P. A. , Erickson, S. K. , & Williams, D. L. (2002). Altered adrenal gland cholesterol metabolism in the apoE‐deficient mouse. Journal of Lipid Research, 43, 1920–1926. 10.1194/jlr.M200205-JLR200 12401891

[gtc13166-bib-0072] Todd, E. V. , Ortega‐Recalde, O. , Liu, H. , Lamm, M. S. , Rutherford, K. M. , Cross, H. , Black, M. A., Kardailsky, O., Marchall Graves, J. A., Hore, T. A., Godwin, J. R., & Gemmell, N. J. (2019). Stress, novel sex genes, and epigenetic reprogramming orchestrate socially controlled sex change. Science Advances, 5, eaaw7006. 10.1126/sciadv.aaw7006 31309157 PMC6620101

[gtc13166-bib-0073] Toga, K. , & Bono, H. (2023). Meta‐analysis of public RNA sequencing data revealed potential key genes associated with reproductive division of labor in social hymenoptera and termites. International Journal of Molecular Sciences, 24, 8353. 10.3390/ijms24098353 37176060 PMC10179490

[gtc13166-bib-0074] Trim Galore . (2022). FelixKrueger. Trim Galore. https://github.com/FelixKrueger/TrimGalore.

[gtc13166-bib-0075] Wang, D. S. , Kobayashi, T. , Zhou, L. Y. , Paul‐Prasanth, B. , Ijiri, S. , Sakai, F. , Okubo, K., Morohashi, K., & Nagahama, Y. (2007). Foxl2 up‐regulates aromatase gene transcription in a female‐specific manner by binding to the promoter as well as interacting with ad4 binding protein/steroidogenic factor 1. Molecular Endocrinology, 21, 712–725. 10.1210/me.2006-0248 17192407

[gtc13166-bib-0076] Wang, X. G. , & Orban, L. (2007). Anti‐Müllerian hormone and 11 β‐hydroxylase show reciprocal expression to that of aromatase in the transforming gonad of zebrafish males. Developmental Dynamics, 236, 1329–1338. 10.1002/dvdy.21129 17393497

[gtc13166-bib-0077] Webster, K. A. , Schach, U. , Ordaz, A. , Steinfeld, J. S. , Draper, B. W. , & Siegfried, K. R. (2017). Dmrt1 is necessary for male sexual development in zebrafish. Developmental Biology, 422, 33–46. 10.1016/j.ydbio.2016.12.008 27940159 PMC5777149

[gtc13166-bib-0078] Wu, D.‐M. , He, Z. , Chen, T. , Liu, Y. , Ma, L.‐P. , & Ping, J. (2016). DNA hypermethylation of acetoacetyl‐CoA synthetase contributes to inhibited cholesterol supply and steroidogenesis in fetal rat adrenals under prenatal nicotine exposure. Toxicology, 340, 43–52. 10.1016/j.tox.2016.01.002 26776438

[gtc13166-bib-0079] Wu, X. , Yang, Y. , Zhong, C. , Guo, Y. , Wei, T. , Li, S. , Lin H., & Liu, X. (2020). Integration of ATAC‐seq and RNA‐seq unravels chromatin accessibility during sex reversal in orange‐spotted grouper (*Epinephelus coioides*). International Journal of Molecular Sciences, 21, 2800. 10.3390/ijms21082800 32316525 PMC7215633

[gtc13166-bib-0080] Xiao, L. , Guo, Y. , Wang, D. , Zhao, M. , Hou, X. , Li, S. , Lin H., & Zhang, Y. (2020). Beta‐hydroxysteroid dehydrogenase genes in orange‐spotted grouper (*Epinephelus coioides*): Genome‐wide identification and expression analysis during sex reversal. Frontiers in Genetics, 11, 161. 10.3389/fgene.2020.00161 32194632 PMC7064643

[gtc13166-bib-0081] Xue, L. , Jia, D. , Xu, L. , Huang, Z. , Fan, H. , Chen, B. , Yang, L., Wang, Z., Li, D., & Gao, Y. (2021). Bulk and single‐cell RNA‐seq reveal the sexually dimorphic expression pattern of dmrtb1 in zig‐zag eel (*Mastacembelus armatus*). Aquaculture, 545, 737194. 10.1016/j.aquaculture.2021.737194

[gtc13166-bib-0082] Zhang, C. , He, Q. , Cheng, H. , Li, L. , Ruan, X. , Duan, X. , Huang, F., Yang, H., Zhang, H., Shi, H., Wang, Q., & Zhao, H. (2022). Transcription factors foxl2 and foxl3 regulate cyp19a1a and cyp11b in orange‐spotted grouper (*Epinephelus coioides*). Aquaculture Reports, 25, 101243. 10.1016/j.aqrep.2022.101243

[gtc13166-bib-0083] Zhang, X. , Guan, G. , Li, M. , Zhu, F. , Liu, Q. , Naruse, K. , Herpin, A., Nagahama, Y., Li, L., & Hong, Y. (2016). Autosomal gsdf acts as a male sex initiator in the fish medaka. Scientific Reports, 6, 19738. 10.1038/srep19738 26813267 PMC4728440

[gtc13166-bib-0084] Zhang, Y. , Zhang, S. , Liu, Z. , Zhang, L. , & Zhang, W. (2013). Epigenetic modifications during sex change repress gonadotropin stimulation of cyp19a1a in a teleost ricefield eel (*Monopterus albus*). Endocrinology, 154, 2881–2890. 10.1210/en.2012-2220 23744638

[gtc13166-bib-0085] Zhao, X. , Luo, M. , Li, Z. , Zhong, P. , Cheng, Y. , Lai, F. , Wang, X., Min, J., Bai, M., Yang, Y., Cheng, H., & Zhou, R. (2018). Chromosome‐scale assembly of the Monopterus genome. GigaScience, 7, giy046. 10.1093/gigascience/giy046 29688346 PMC5946948

[gtc13166-bib-0086] Zhou, Y. , Zhou, B. , Pache, L. , Chang, M. , Khodabakhshi, A. H. , Tanaseichuk, O. , Benner, C., & Chanda, S. K. (2019). Metascape provides a biologist‐oriented resource for the analysis of systems‐level datasets. Nature Communications, 10, 1523. 10.1038/s41467-019-09234-6 PMC6447622 30944313

[gtc13166-bib-0087] Zhu, B.‐H. , Xiao, J. , Xue, W. , Xu, G.‐C. , Sun, M.‐Y. , & Li, J.‐T. (2018). P_RNA_scaffolder: a fast and accurate genome scaffolder using paired‐end RNA‐sequencing reads. BMC Genomics, 19, 175. 10.1186/s12864-018-4567-3 29499650 PMC5834899

[gtc13166-bib-0088] Zhu, Y. , Wang, C. , Chen, X. , & Guan, G. (2016). Identification of gonadal soma‐derived factor involvement in *Monopterus albus* (protogynous rice field eel) sex change. Molecular Biology Reports, 43, 629–637. 10.1007/s11033-016-3997-8 27230579

[gtc13166-bib-0089] Zou, Q. , Shen, W. , Le, S. , Li, Y. , & Hu, F. (2016). SeqKit: A cross‐platform and ultrafast toolkit for FASTA/Q file manipulation. PLoS One, 11, e0163962. 10.1371/journal.pone.0163962 27706213 PMC5051824

