# Supplementary material for: Meta‐analysis of gonadal transcriptome provides novel insights into sex change mechanism across protogynous fishes
Source: Genes Cells. 2024 Sep 29;29(11):1052–68. doi: 10.1111/gtc.13166 (PMC11555629; doi:10.1111/gtc.13166)
Supplement: Supplementary file 1 — Figure S1. Schematic diagram of the analysis pipeline in this study. The consistent line style of the arrows indicates the utilization of common tools for conducting data processing. The letters in parentheses next to the species names represent the following order names: L, Labriformes; P, Perciformes; S, Synbranchiformes. Figure S2. Hypothetical ovarian steroid hormones biosynthesis pathway in protogynous fishes. The pathway was visualized using PathVisio (https://pathvisio.org), referencing several fish cholesterol synthesis pathways. Gene products are represented in black text, while metabolites are in blue text. Gene product annotations follow Ensembl Zebrafish gene symbols. Red‐filled boxes represent gene products with confirmed upregulated expression in this study, while blue‐filled boxes denote gene products with downregulated expression. Open boxes represent gene products with no expression variation. Light green‐filled boxes represent gene products not included among the 7289 factors but confirmed to be present in at least the three‐spot wrasse transcriptome. Dark gray‐filled boxes indicate gene products that could not be identified within the three‐spot wrasse transcriptome. Circles extending from gene products represent catalytic reactions, while black arrows indicate the conversion of metabolites. Figure S3. Histological sections of control ovaries and transitional gonads treated AI for 7 days in the threespot wrasse, Halichoeres trimaculatus. Figure S4. Principal component analysis (PCA) results of gene expression data from each species used in this study. The status of transitional gonads is indicated according to the metadata descriptions in the Supplementary Table 1. The black line circles in Monopterus albus represent data derived from different bioprojects. The analysis and visualization were performed using the R program (version 4.4.0) with the ‘pcaExplorer’ package (version 2.30.0; Marini & Binder, 2019). [file GTC-29-1052-s001.pdf]

Supplementary materials

Meta-analysis of gonadal transcriptome provides novel insights into sex change mechanism across protogynous fishes

Ryo Nozu[1,2], Mitsutaka Kadota[3,4], Masaru Nakamura[5], Shigehiro Kuraku[6,7], Hidemasa Bono[1,2*]

[1] Laboratory of Genome Informatics, Graduate School of Integrated Sciences for Life, Hiroshima University, 3-10-23 Kagamiyama, Higashi-Hiroshima, Hiroshima 739-0046, Japan

[2] Laboratory of BioDX, Genome Editing Innovation Center, Hiroshima University, Hiroshima, Japan

[3] Laboratory for Phyloinformatics, RIKEN Center for Biosystems Dynamics Research (BDR), Kobe, Japan

[4] Laboratory for Developmental Genome System, RIKEN Center for Biosystems Dynamics Research (BDR), Kobe, Japan

[5] Okinawa Churashima Research Center, Okinawa Churashima Foundation, Motobu-cho, Okinawa, Japan

[6] Molecular Life History Laboratory, Department of Genomics and Evolutionary Biology, National Institute of Genetics, Mishima, Japan

[7] Department of Genetics, Sokendai (Graduate University for Advanced Studies), Mishima, Japan.

Supplementary figure 1.

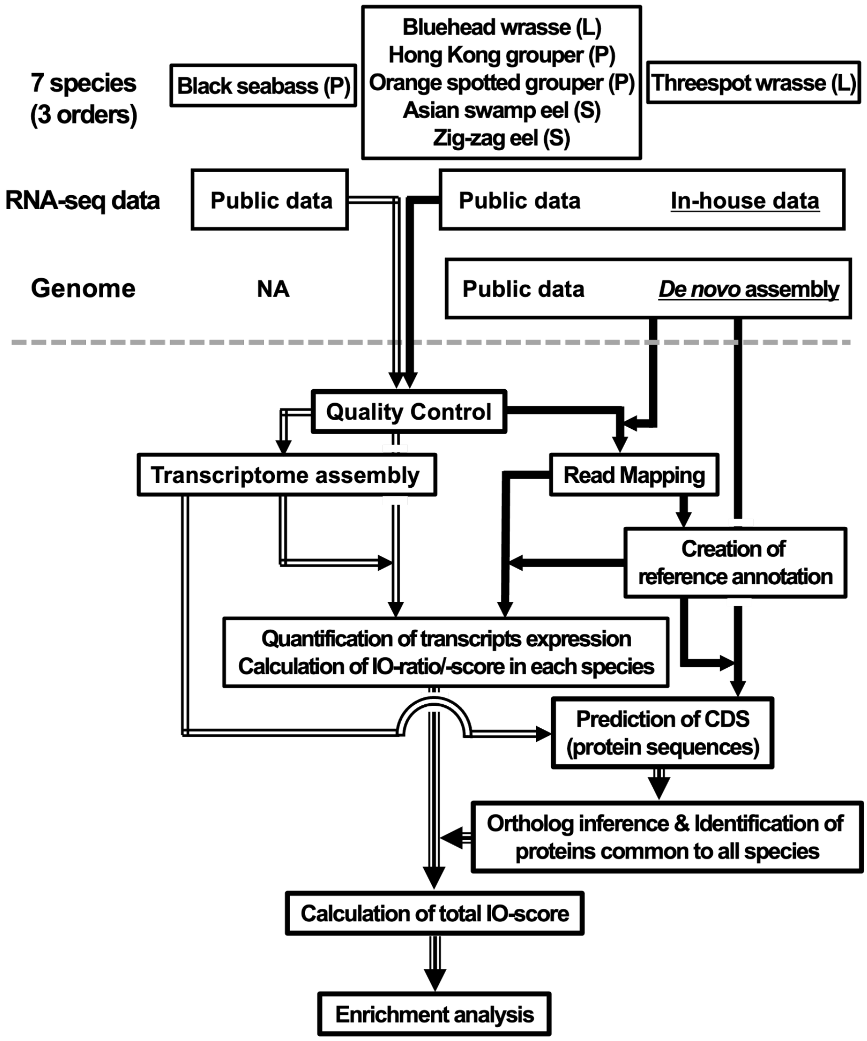

Figure S1. Schematic diagram of the analysis pipeline in this study. The consistent line style of the arrows indicates the utilization of common tools for conducting data processing. The letters in parentheses next to the species names represent the following order names; L: Labriformes, P: Perciformes, S: Synbranchiformes.

Supplementary figure 2.

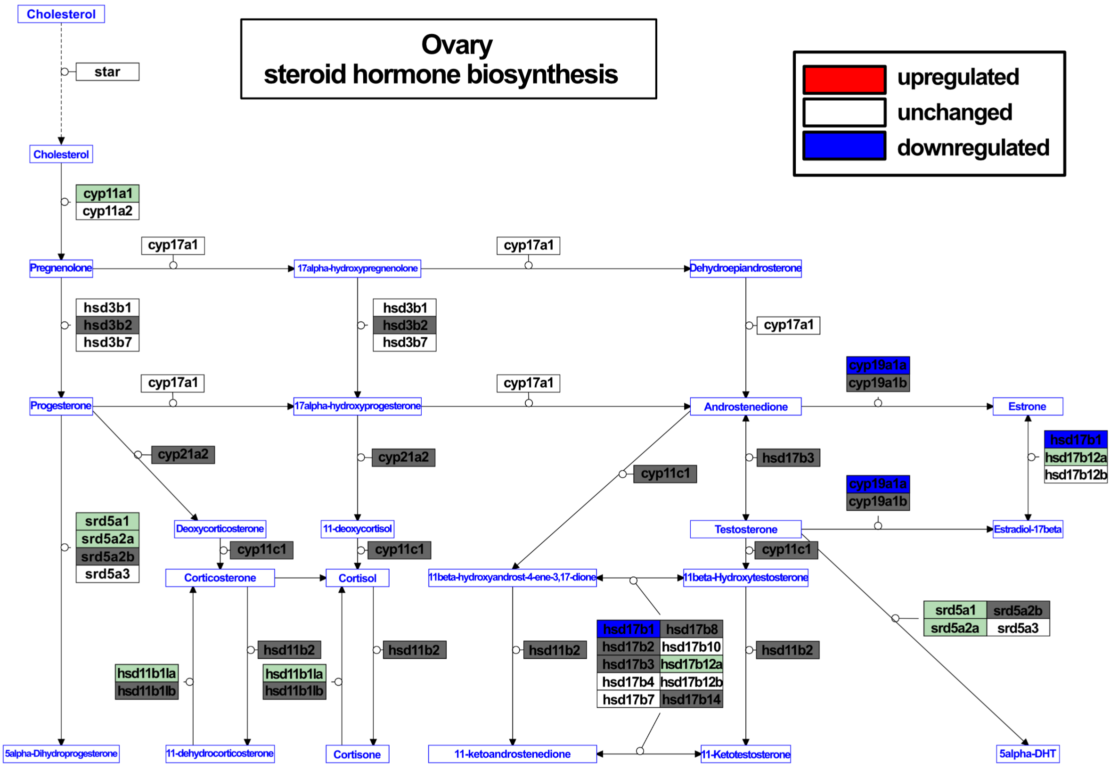

Figure S2. Hypothetical ovarian steroid hormones biosynthesis pathway in protogynous fishes. The pathway was visualized using PathVisio (https://pathvisio.org), referencing several fish cholesterol synthesis pathways. Gene products are represented in black text, while metabolites are in blue text. Gene product annotations follow Ensembl Zebrafish gene symbols. Red-filled boxes represent gene products with confirmed upregulated expression in this study, while blue-filled boxes denote gene products with downregulated expression. Open boxes represent gene products with no expression variation. Light green-filled boxes represent gene products not included among the 7289 factors but confirmed to be present in at least the three-spot wrasse transcriptome. Dark gray-filled boxes indicate gene products that could not be identified within the three-spot wrasse transcriptome. Circles extending from gene products represent catalytic reactions, while black arrows indicate the conversion of metabolites.

Supplementary figure 3.

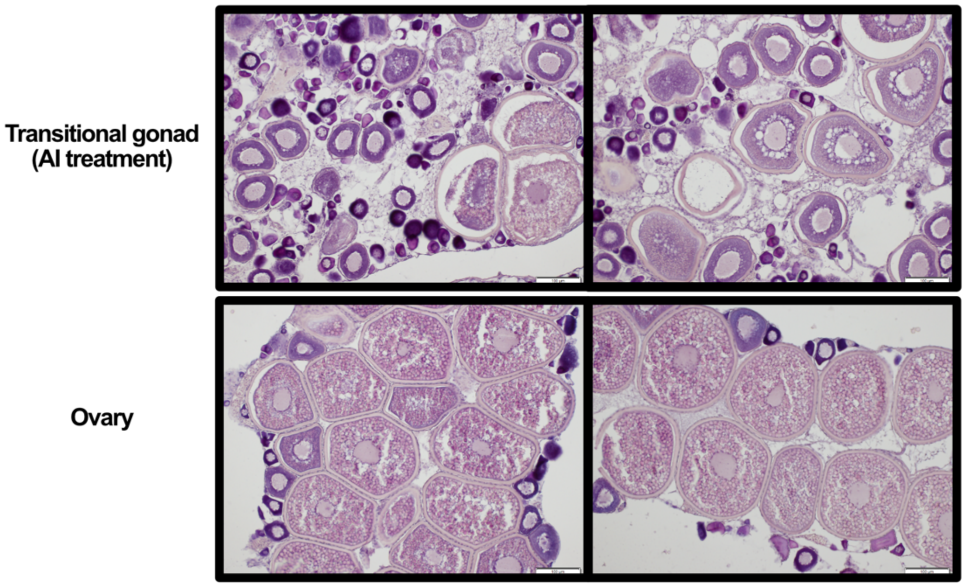

Figure S3. Histological sections of control ovaries and transitional gonads treated AI for 7 days in the threespot wrasse, *Halichoeres trimaculatus*.

Supplementary figure 4.

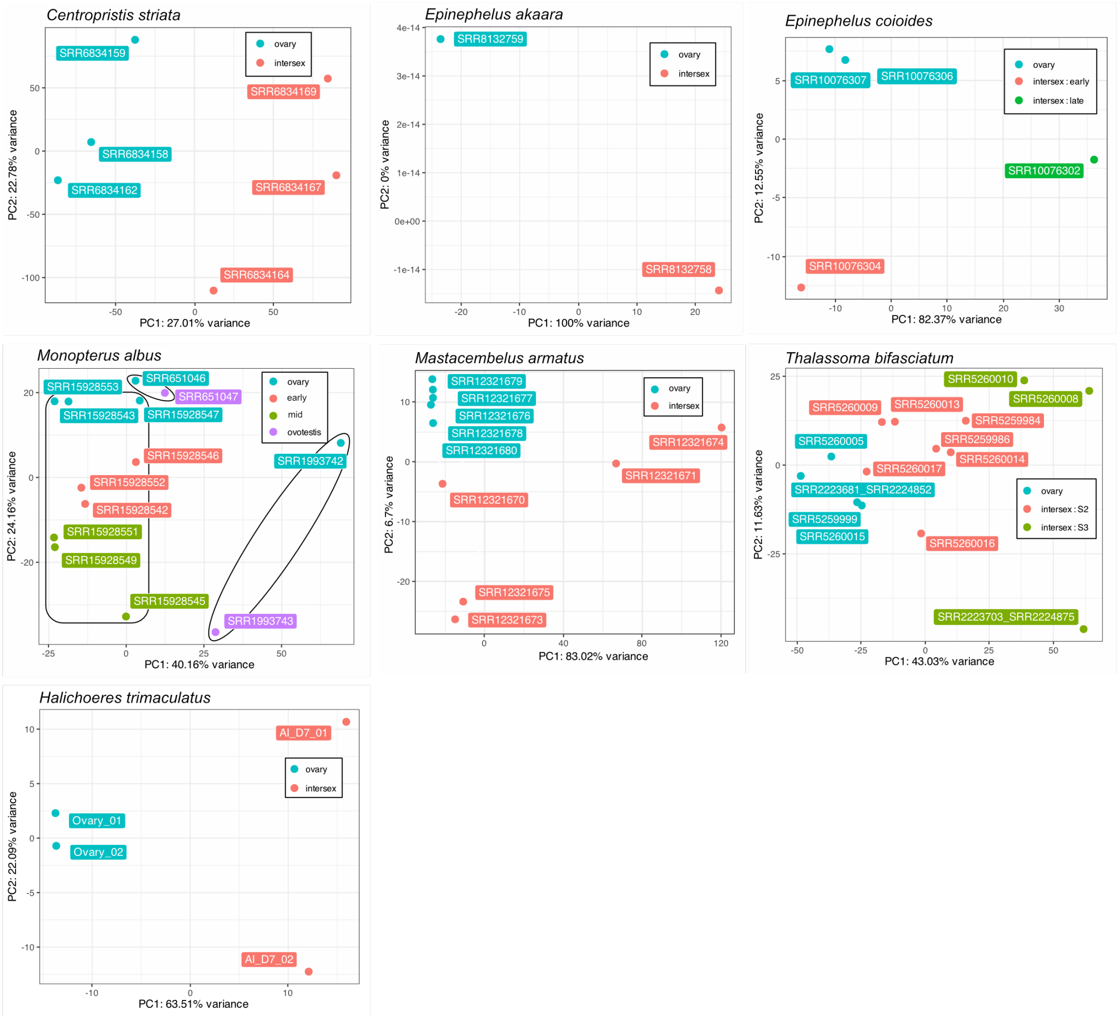

Figure S4. Principal component analysis (PCA) results of gene expression data from each species used in this study. The status of transitional gonads is indicated according to the metadata descriptions in the supplementary table 1. The black line circles in *Monopterus albus* represent data derived from different bioprojects. The analysis and visualization were performed using the R program (version 4.4.0) with the 'pcaExplorer' package (version 2.30.0; Marini and Binder, 2019).
